# Trajectory of long covid symptoms after covid-19 vaccination: community based cohort study

**DOI:** 10.1136/bmj-2021-069676

**Published:** 2022-05-18

**Authors:** Daniel Ayoubkhani, Charlotte Bermingham, Koen B Pouwels, Myer Glickman, Vahé Nafilyan, Francesco Zaccardi, Kamlesh Khunti, Nisreen A Alwan, A Sarah Walker

**Affiliations:** 1 Health Analysis and Life Events Division, Office for National Statistics, Newport, UK; 2 Leicester Real World Evidence Unit, Diabetes Research Centre, University of Leicester, Leicester, UK; 3 National Institute for Health Research Health Protection Research Unit in Healthcare Associated Infections and Antimicrobial Resistance, University of Oxford, Oxford, UK; 4 Health Economics Research Centre, Nuffield Department of Population Health, University of Oxford, Oxford, UK; 5 Faculty of Public Health, Environment, and Society, London School of Hygiene and Tropical Medicine, London, UK; 6 School of Primary Care, Population Sciences and Medical Education, Faculty of Medicine, University of Southampton, Southampton, UK; 7 NIHR Southampton Biomedical Research Centre, University of Southampton and University Hospital Southampton NHS Foundation Trust, Southampton, UK; 8 NIHR Applied Research Collaboration (ARC) Wessex, Southampton, UK; 9 Nuffield Department of Medicine, University of Oxford, Oxford, UK

## Abstract

**Objective:**

To estimate associations between covid-19 vaccination and long covid symptoms in adults with SARS-CoV-2 infection before vaccination.

**Design:**

Observational cohort study.

**Setting:**

Community dwelling population, UK.

**Participants:**

28 356 participants in the Office for National Statistics COVID-19 Infection Survey aged 18-69 years who received at least one dose of an adenovirus vector or mRNA covid-19 vaccine after testing positive for SARS-CoV-2 infection.

**Main outcome measure:**

Presence of long covid symptoms at least 12 weeks after infection over the follow-up period 3 February to 5 September 2021.

**Results:**

Mean age of participants was 46 years, 55.6% (n=15 760) were women, and 88.7% (n=25 141) were of white ethnicity. Median follow-up was 141 days from first vaccination (among all participants) and 67 days from second vaccination (83.8% of participants). 6729 participants (23.7%) reported long covid symptoms of any severity at least once during follow-up. A first vaccine dose was associated with an initial 12.8% decrease (95% confidence interval −18.6% to −6.6%, P<0.001) in the odds of long covid, with subsequent data compatible with both increases and decreases in the trajectory (0.3% per week, 95% confidence interval −0.6% to 1.2% per week, P=0.51). A second dose was associated with an initial 8.8% decrease (95% confidence interval −14.1% to −3.1%, P=0.003) in the odds of long covid, with a subsequent decrease by 0.8% per week (−1.2% to −0.4% per week, P<0.001). Heterogeneity was not found in associations between vaccination and long covid by sociodemographic characteristics, health status, hospital admission with acute covid-19, vaccine type (adenovirus vector or mRNA), or duration from SARS-CoV-2 infection to vaccination.

**Conclusions:**

The likelihood of long covid symptoms was observed to decrease after covid-19 vaccination and evidence suggested sustained improvement after a second dose, at least over the median follow-up of 67 days. Vaccination may contribute to a reduction in the population health burden of long covid, although longer follow-up is needed.

## Introduction

By the end of 2021 in the UK, when 90% of the population had received at least one dose of a covid-19 vaccine, nearly 14 million cases of SARS-CoV-2 had been confirmed, 640 000 patients had been admitted to hospital, and 158 000 had died with covid-19.^1^ Symptoms of infection may persist for months, defined in UK clinical guidelines as ongoing symptomatic covid-19 (signs and symptoms 4-12 weeks after onset) or post-covid-19 syndrome (>12 weeks after onset).^2^ These symptoms are collectively and commonly referred to as long covid. Long covid is characterised by a range of manifestations across organ systems, including fatigue, shortness of breath, and cognitive impairment,^3^ often with fluctuating periods of wellness followed by relapse.^4^ ^5^ ^6^ By 8 February 2021, nearly 6% of adults in England might have experienced prolonged symptoms after SARS-CoV-2 infection since the pandemic began,^7^ and in October 2021 an estimated 1.2 million people in private households in the UK (1.9%) had reported experiencing long covid, with symptoms in two thirds of these individuals having a detrimental impact on day-to-day activities.^3^

Population level immunisation against covid-19 began in the UK on 8 December 2020, and both adenovirus vector and mRNA vaccines administered to the population have shown safety and efficacy in trials^8^ ^9^ ^10^ ^11^ and real world effectiveness at reducing rates of SARS-CoV-2 infection,^12^ ^13^ transmission,^14^ admission to hospital,^15^ and death.^15^ ^16^ Preliminary research suggests that long covid symptoms are less common in breakthrough infections,^17^ but the effectiveness of vaccination on pre-existing long covid is less clear. Anecdotal evidence suggests variations in the lived experience of long covid after vaccination, with patients describing improvement, deterioration, and no change in their symptoms. In an online survey of members of a long covid patient advocacy group in the US, about 40% of respondents reported full or partial symptom resolution after vaccination and 14% reported deterioration.^18^ In a similar survey conducted by a UK based patient group, more than a half of participants experienced an improvement in long covid symptoms and a fifth experienced a worsening of symptoms.^19^ Although such studies are informative, they included self-selected groups of participants who might not be representative of the population of interest and lacked control groups and long term follow-up, and other studies have included small sample sizes.^20^ ^21^ A quarter of the UK population aged 12 years and older were yet to receive two doses of a covid-19 vaccine by 5 September 2021, and 16% had not received their first dose.^1^ Possible vaccine hesitancy among people with long covid symptoms has been identified through social media discourse.^22^

Greater evidence is therefore needed on the symptomology of SARS-CoV-2 infection after vaccination, which may facilitate informed decision making among individuals with long covid. To estimate associations between covid-19 vaccination and long covid symptoms in adults infected with SARS-CoV-2 before vaccination, we used data from the Office for National Statistics COVID-19 Infection Survey, a large, community based population survey.

## Methods

### Study data and design

Data were obtained from the COVID-19 Infection Survey,^23^ a longitudinal survey of people aged 2 years or older in randomly sampled UK households (excluding communal establishments such as hospitals, care homes, halls of residence, and prisons).

Enrolment rates were as high as 51% in the initial pilot phase of the survey from April 2020, when eligible households comprised previous respondents to ONS surveys who had consented to participate in future research. As the sample was expanded and transitioned to random selection from address lists in August 2020, however, the enrolment rate dropped to 12% (see supplementary table 1 for details). Once participants are enrolled into the study, the attrition rate is generally low; using a definition of either formally withdrawing from the study or having not attended the three most recently scheduled follow-up visits, the attrition rate among enrolled survey participants was less than 1% in 2021.

A study worker visited each selected household, after verbal agreement to participate had been obtained, to provide written confirmed consent (from parents or carers for those aged 2-15 years; those aged 10-15 years also provided written assent). At the first visit, participants could consent for (optional) follow-up visits every week for the next month and then monthly for 12 months or longer.

All participants provided a self-collected nose and throat swab sample for reverse transcription polymerase chain reaction testing at each follow-up visit. Those aged 16 years or older in a random subsample of households (initially 10% but expanded from April 2021), and those in households where another household member previously tested positive for SARS-CoV-2, were invited to provide monthly blood samples for S antibody testing. Participants also reported whether they had tested positive either for SARS-CoV-2 or for antibodies to SARS-CoV-2 outside of the study (for example, through national testing programmes).

At every monthly visit from 3 February 2021, survey participants were asked whether they would describe themselves as currently experiencing long covid—defined as symptoms persisting for at least four weeks from confirmed or suspected SARS-CoV-2 infection that could not be explained by another health condition. This definition uses self-classification of long covid rather than a prespecified symptoms list or clinical diagnosis, and thus reflects participants’ perception of whether their lived experience is consistent with what they understand of the condition. Participants who responded positively to the long covid question were further asked about the extent to which their day-to-day activities were limited as a result and the presence of 21 individual symptoms as part of their experience of long covid (selected on the basis of being among the most commonly reported when the survey question was developed^5^ ^6^ ^24^; see full list in supplementary table 2).

For participants in England, information on vaccination (number of doses, dates, manufacturer) was obtained from self-reported responses to the COVID-19 Infection Survey and linked National Immunisation Management System records, with the latter being prioritised when data conflicted. Concordance between self-reported and National Immunisation Management System data was previously found to be high for vaccination type (98%) and date (95% within ±7 days).^12^ As administrative records were not available for participants in Wales, Scotland, and Northern Ireland, vaccination data for these individuals were taken from the survey alone.

### Inclusion and exclusion criteria

The analysis included survey participants aged 18 to 69 years on 3 February 2021. Participants were included if they responded to the survey question on long covid at least once by 5 September 2021 (end of follow-up), received at least one dose of a covid-19 vaccine before or during the follow-up period, and received a positive swab or blood test result for SARS-CoV-2, either through the survey or reported outside of the study, before vaccination. We excluded survey participants who remained unvaccinated by 5 September 2021 because they were likely to differ from those who were vaccinated according to unmeasured characteristics (for example, personal considerations related to vaccine hesitancy).

### Infection date

We defined the time of infection as the date of a first positive swab or antibody test result (ignoring blood test results after first vaccination), or the date when participants first thought they had covid-19 that was later confirmed by a positive test result, whichever was earlier. Although the survey question asks about long covid symptoms persisting for at least four weeks from infection, for this analysis we used a longer 12 week threshold, consistent with the UK clinical case definition of post-covid-19 syndrome^2^ and the World Health Organization’s definition of post-covid-19 condition.^25^ We therefore excluded any follow-up visits within 12 weeks of the infection date.

### Follow-up

Participants were observed from their first survey follow-up visit that took place after their first SARS-CoV-2 infection and after the long covid question was added to the COVID-19 Infection Survey on 3 February 2021. Follow-up ended on the date of the participants’ final follow-up visit that took place by 5 September 2021.

### Vaccination variables

The explanatory variables of interest were first and second doses of an adenovirus vector (ChAdOx1 nCoV-19 (AZD1222), Oxford-AstraZeneca) or mRNA (BNT162b2, Pfizer-BioNTech; mRNA-1273, Moderna) covid-19 vaccine. The recommended interval between the first and second vaccine doses was 11 to 12 weeks for most participants in the study sample (having been increased from four weeks on 30 December 2020), with a reduction to eight weeks in May 2021 for people in the top nine vaccination priority groups.^26^ For each vaccine dose, we estimated the associated change in outcomes using a binary variable to indicate whether participants had received each dose at each follow-up visit; and a variable equal to the number of days since receiving each dose at each follow-up visit to estimate post-vaccination changes in the outcome trajectory (set to 0 for visits before receiving each dose). This specification implies that any change in the odds of long covid occurs instantly after vaccination, although in reality this may take place over several days or weeks.

### Outcomes

The primary outcome at each visit was long covid of any severity, with a secondary outcome of long covid resulting in limitation of day-to-day activities (“a little” or “a lot” versus “not at all” or no long covid); this definition of functional impairment is standardised across data collections by the UK Government Statistical Service and is designed to measure disability as defined in the Equality Act 2010. We also evaluated the 10 individual symptoms that were most commonly reported over the follow-up period and whether participants were experiencing more than three or more than five of the 21 symptoms included on the survey.

### Covariates

As well as time from infection and the vaccination variables to modify the time trajectory of long covid, we adjusted for covariates hypothesised to be related to vaccine type and timing^27^ and the probability of experiencing long covid symptoms^3^: age, sex, white or non-white ethnicity, region or country, area deprivation fifth group, health status, patient-facing health or social care worker, and hospital admission with acute covid-19. We also adjusted for calendar time of infection to control for temporal effects that might be related to the risk of developing prolonged symptoms, such as viral variant (the alpha and delta variants were both dominant at different periods during follow-up) and changes in healthcare practice. Supplementary table 3 provides details of the covariates.

### Statistical analysis

We compared covariates between participants who received an adenovirus vector vaccine and mRNA vaccine using means and proportions for continuous and categorical variables, respectively. Standardised differences >10% indicated large differences.^28^

An individual level interrupted time series approach was used to estimate associations between vaccination variables and outcomes.^29^ For each outcome, we included all vaccination variables and covariates in a binary logistic regression model and estimated robust (clustered) standard errors to account for correlation within participants from having repeated measures. We opted for a linear fit for time since infection as this specification minimised the bayesian information criterion compared with higher order polynomial or spline fits, thus providing a better balance between goodness of fit and parsimony (see supplementary figure 1).

We explored heterogeneity in associations between vaccination and long covid by interacting all four vaccination variables (change in level and slope after each dose) with each of age group (18-29 years, 30-39 years, 40-49 years, 50-59 years, ≥60 years), sex, white or non-white ethnicity, area deprivation fifth group, health status, hospital admission with acute covid-19, vaccine type (adenovirus vector or mRNA), and duration from infection to first vaccination (modelled as a restricted cubic spline). For each outcome, statistically significant interactions were identified at the 5% level after performing Holm-Bonferroni and Benjamini-Yekutieli corrections to P values to account for multiple comparisons across vaccination variables and modifiers. All statistical analyses were performed using R version 3.6.

### Sensitivity analyses

We restricted the sample firstly to participants with at least one observation before and after each vaccination and secondly to those with at least three observations after each vaccination. We omitted follow-up visits within the first week after each vaccination, which may have been influenced by post-vaccine side effects. Survey participants were added who remained unvaccinated by their last follow-up visit during the study period (who were excluded from the main analysis). Because mass testing for SARS-CoV-2 was largely unavailable during the first wave of the pandemic, we excluded participants who were infected before the start of the second wave on 11 September 2020,^30^ and therefore these infections were likely to have been more severe than the majority included in the analysis. For 2.5% of participants where this was determined we reset the infection date by when the participant first thought they had covid-19 (later confirmed by a positive test result) that was >14 days before a positive swab result (the estimated maximum incubation period^31^); these participants may have been reinfected but only their second infection was validated by means of a positive test result, so the infection date was moved forward to the date of this test. Finally, we excluded participants whose infection date was determined by a positive blood test result for SARS-CoV-2 antibodies that was obtained before or on the date of their first survey follow-up visit; the precise timing of infection was unknown for these participants.

### Patient and public involvement

NAA contributed to this paper both as someone with lived experience of long covid and as a public health researcher. She has previously strongly advocated against the separation of identities of people with long covid who are also scientists, researchers, or health professionals. She has also written on patient involvement in long covid research and the lessons learnt that could apply to other conditions.^32^ ^33^ She contributed to informing this analysis’s concept, design, and interpretation.

Although we did not directly involve patients and members of the public more broadly, the study design was informed by views expressed by patient representatives in monthly meetings attended by DA (the Department of Health and Social Care’s long covid ministerial roundtable, NHS England’s long covid national taskforce). These meetings were attended by the founders of three major long covid patient support groups in the UK, whose insights on aspects such as the range of long covid symptoms experienced and their relapsing and remitting nature informed the data collected and definitions used in this study.

## Results

**Fig 1:**
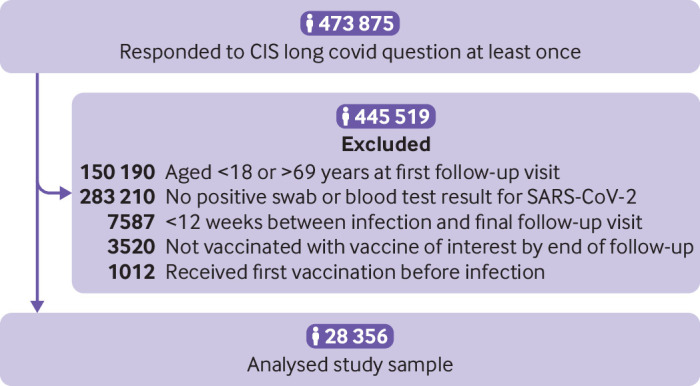
Study participant flow diagram. CIS=Office for National Statistics COVID-19 Infection Survey

Of 323 685 participants in the COVID-19 Infection Survey aged 18 to 69 years with at least one visit between 3 February and 5 September 2021, 28 356 had test confirmed SARS-CoV-2 at least 12 weeks before their final visit and had been vaccinated post-infection and were therefore included in analysis (fig 1).

Median time to the final follow-up visit was 169 (interquartile range 141-185) days from the first visit and 267 (219-431) days from first SARS-CoV-2 infection. By design, all study participants received their first vaccination by 5 September 2021, 12 971 (45.7%) after the start of the study period on 3 February. Overall, 23 753 (83.8%) participants were double vaccinated by 5 September 2021, with 20 335 (71.7%) receiving their second dose after 3 February, with a median time between doses of 72 (interquartile range 61-77) days (see supplementary figure 2). Supplementary table 4 shows vaccination status during follow-up by age and health status (two of the main vaccination prioritisation determinants). Participants had a median of 4 (interquartile range 2-5) visits over a median of 141 (interquartile range 86-173) days after their first dose and, among those double vaccinated, 2 (1-3) visits over 67 (20-99) days after their second dose.

At the last visit, the mean age of participants was 46 years (standard deviation 14 years), 55.6% (n=15 760) were women, and 88.7% (n=25 141) were of white ethnicity (table 1). Compared with participants who received an adenovirus vector vaccine, those who received an mRNA vaccine were on average younger (mean 40 *v* 51 years) and more likely to be of non-white ethnicity (13.7% *v* 9.4%), resident in London (27.0% *v* 22.4%) or Northern Ireland (3.3% *v* 1.5%), and a patient-facing health or social care worker (17.1% *v* 6.4%).

**Table 1:** Characteristics of study participants at their final follow-up visit, stratified by covid-19 vaccine type

Characteristics	Full sample (n=28 356)	mRNA vaccine (n=12 859)	Adenovirus vector vaccine (n=15 497)	Standardised difference (%)
Mean (SD) time since SARS-CoV-2 infection (days)	308.9 (129.0)	314.9 (132.9)	304.0 (125.4)	8.4
Mean (SD) time since first covid-19 vaccine dose (days)	130.7 (55.9)	118.0 (67.8)	141.2 (40.7)	−41.5
Mean (SD) age (years)	45.9 (13.6)	40.1 (14.1)	50.7 (11.1)	−83.4
Sex:				
Men	12 596 (44.4)	5466 (42.5)	7130 (46.0)	−7.1
Women	15 760 (55.6)	7393 (57.5)	8367 (54.0)	7.1
Ethnicity*:				
White	25 141 (88.7)	11 097 (86.3)	14 044 (90.6)	−13.6
Non-white	3215 (11.3)	1762 (13.7)	1453 (9.4)	13.6
Region or country:				
Northeast England	1133 (4.0)	524 (4.1)	609 (3.9)	0.7
Northwest England	3990 (14.1)	1774 (13.8)	2216 (14.3)	−1.4
Yorkshire and Humber	2430 (8.6)	1028 (8.0)	1402 (9.0)	−3.8
East Midlands	1755 (6.2)	710 (5.5)	1045 (6.7)	−5.1
West Midlands	2204 (7.8)	917 (7.1)	1287 (8.3)	−4.4
East of England	2447 (8.6)	1044 (8.1)	1403 (9.1)	−3.3
London	6942 (24.5)	3470 (27.0)	3472 (22.4)	10.6
Southeast England	2919 (10.3)	1208 (9.4)	1711 (11.0)	−5.4
Southwest England	1276 (4.5)	580 (4.5)	696 (4.5)	0.1
Northern Ireland	657 (2.3)	421 (3.3)	236 (1.5)	11.5
Scotland	1376 (4.9)	620 (4.8)	756 (4.9)	−0.3
Wales	1227 (4.3)	563 (4.4)	664 (4.3)	0.5
Area deprivation fifth group†:				
1 (most deprived)	3825 (13.5)	1779 (13.8)	2046 (13.2)	1.8
2	5392 (19.0)	2671 (20.8)	2721 (17.6)	8.2
3	5857 (20.7)	2633 (20.5)	3224 (20.8)	−0.8
4	6474 (22.8)	2914 (22.7)	3560 (23.0)	−0.7
5 (least deprived)	6808 (24.0)	2862 (22.3)	3946 (25.5)	−7.5
Patient-facing health or social care worker	3190 (11.2)	2198 (17.1)	992 (6.4)	33.7
Health conditions‡	3851 (13.6)	1531 (11.9)	2320 (15.0)	−9.0
Hospital admission with acute covid-19§	900 (3.2)	359 (2.8)	541 (3.5)	−4.0

SD=standard deviation.

* The study sample size did not permit disaggregation of ethnicity beyond white and non-white groups.

† Area deprivation was based on English indices of deprivation 2019, the Welsh index of multiple deprivation 2019, the Scottish index of multiple deprivation 2020, and the Northern Ireland multiple deprivation measure 2017.

‡ Health conditions were self-reported rather than clinically diagnosed based on the survey question: “Do you have any physical or mental health conditions or illnesses lasting or expected to last 12 months or more (excluding any long-lasting covid-19 symptoms)?”

§ Hospital admission with acute covid-19 was self-reported rather than derived from medical records.

### Long covid trajectories before and after vaccination

**Fig 2:**
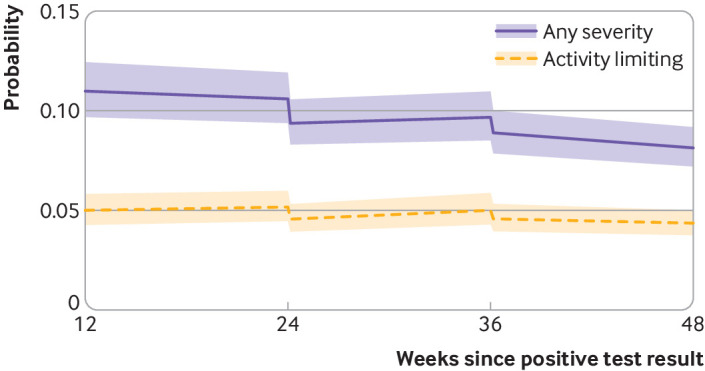
Modelled probabilities of long covid for a hypothetical study participant who received a first covid-19 vaccine dose 24 weeks after SARS-CoV-2 infection and a second dose 12 weeks later. Probabilities are shown for participants of mean age (50 years) and in the modal group for other covariates (woman, white ethnicity, resident in London, resident in an area in the least deprived fifth group, not a patient-facing health or social care worker, no pre-existing health conditions, not admitted to hospital during the acute phase of infection, infected on 7 September 2020). Although estimated probabilities are specific to this profile, proportional changes in probabilities after vaccination do not vary across characteristics and can therefore be generalised to other profiles. Dashed lines represent timing of vaccination. Shaded areas are 95% confidence intervals

Long covid symptoms of any severity were reported by 6729 participants (23.7%) at least once during follow-up. Before vaccination, the odds of experiencing long covid changed little over time (−0.3% per week, 95% confidence interval −0.9% to 0.2%, P=0.25; table 2). A first vaccine dose was associated with an initial 12.8% decrease (95% confidence interval −18.6% to −6.6%, P<0.001) in the odds, with the data being compatible with both increases and decreases in the trajectory (0.3% per week, 95% confidence interval −0.6% to 1.2% per week, P=0.51) between the first and second doses. A second vaccine dose was associated with an initial 8.8% decrease (−14.1% to −3.1%, P=0.003) in the odds, followed by a decrease of 0.8% (−1.2% to −0.4%, P<0.001) per week (fig 2).

**Table 2:** Estimated time trajectories of long covid from SARS-CoV-2 infection, and changes in trajectories after covid-19 vaccination

Outcomes	Estimate (SE)*	P value	Odds ratio† (95% CI)
**Long covid of any severity**			
Time trajectory (per week)	−0.003 (0.003)	0.25	0.997 (0.991 to 1.002)
First vaccine dose (change in level)	−0.137 (0.035)	<0.001	0.872 (0.814 to 0.934)
Second vaccine dose (change in level)	−0.092 (0.031)	0.003	0.912 (0.859 to 0.969)
Time since first vaccination (per week)	0.006 (0.005)	0.21	1.006 (0.996 to 1.016)
Time since second vaccination (per week)	−0.011 (0.005)	0.03	0.989 (0.979 to 0.999)
**Activity-limiting long covid**			
Time trajectory (per week)	0.003 (0.004)	0.44	1.003 (0.996 to 1.010)
First vaccine dose (change in level)	−0.131 (0.044)	0.003	0.877 (0.805 to 0.955)
Second vaccine dose (change in level)	−0.096 (0.038)	0.01	0.909 (0.844 to 0.979)
Time since first vaccination (per week)	0.006 (0.006)	0.35	1.006 (0.994 to 1.018)
Time since second vaccination (per week)	−0.013 (0.006)	0.03	0.987 (0.976 to 0.998)

CI=confidence interval; SE=standard error.

Estimates and odds ratios are adjusted for age, sex, white or non-white ethnicity, region or country, area deprivation fifth group, health status, patient-facing health or social care worker, hospital admission with acute covid-19, and calendar time of infection.

* Estimates and standard errors are on the logit scale.

† Odds ratios for time since first or second vaccination represent modification of the time trajectory.

Long covid resulting in limitation of activities was reported by 4747 participants (16.7%) at least once during follow-up. A first vaccine dose was associated with an initial 12.3% decrease (−19.5% to −4.5%, P=0.003) in the odds of activity-limiting long covid, followed by an uncertain trajectory (0.9% per week, 95% confidence interval −0.2% to 1.9%, P=0.11) until the second dose was administered. A second vaccine dose was associated with an initial 9.1% decrease (95% confidence interval −15.6% to −2.1%, P=0.01) in the odds of activity-limiting long covid, followed by −0.5% per week (95% confidence interval −1.0% to 0.05%, P=0.08) until the end of follow-up.

To illustrate the impact of each vaccine dose, figure 2 shows the estimated probability of reporting long covid for study participants receiving their first vaccine dose 24 weeks after infection and their second dose 12 weeks later. Sensitivity analyses (see supplementary figures 3a-i) were generally consistent with the main results. Evidence of a change to an increasing trend in long covid between first and second vaccine doses was, however, stronger when the sample was restricted to participants who received their first dose during the follow-up period 3 February to 5 September 2021 (P<0.001 for long covid of any severity, P=0.01 for activity-limiting long covid).

### Heterogeneity by vaccine type, duration since infection, and participant characteristics

**Fig 3:**
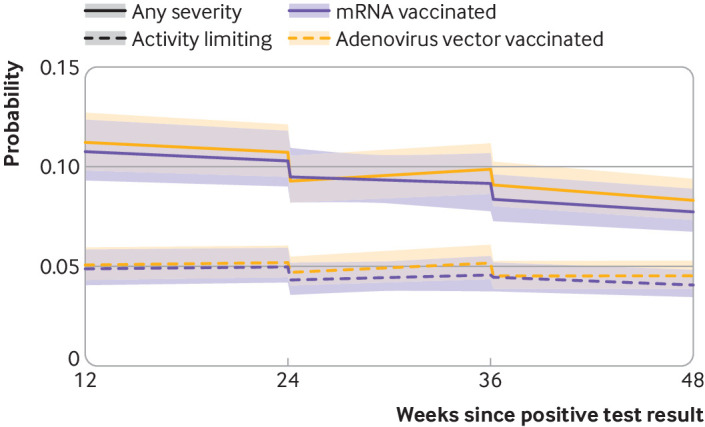
Modelled probabilities of long covid for a hypothetical study participant who received a first dose of an adenovirus vector or mRNA vaccine 24 weeks after SARS-CoV-2 infection and a second dose 12 weeks later. Probabilities are shown for participants of mean age (50 years) and in the modal group for other covariates (woman, white ethnicity, resident in London, resident in an area in the least deprived fifth group, not a patient-facing health or social care worker, no pre-existing health conditions, not admitted to hospital during the acute phase of infection, infected on 7 September 2020). Although estimated probabilities are specific to this profile, proportional changes in probabilities after vaccination do not vary across characteristics and can therefore be generalised to other profiles. Dashed lines represent timing of vaccination. Shaded areas are 95% confidence intervals

We found no statistical evidence of differences in post-vaccination long covid trajectories between participants who received an adenovirus vector vaccine and those who received an mRNA vaccine (table 3, fig 3) for changes in either levels (P=0.31 for dose 1, P=0.97 for dose 2) or slopes (P=0.33 for change between dose 1 and dose 2, and P=0.33 for change after dose 2). Vaccination was associated with an initial 14.9% decrease (95% confidence interval −21.8% to −7.5%, P<0.001) in the odds of long covid after a first dose of an adenovirus vector vaccine, and a numerical 8.9% decrease (95% confidence interval −18.2% to 1.4%, P=0.09) after a first dose of an mRNA vaccine, although the data were also compatible with increased odds for the latter. Decreases in the odds after a second vaccine dose were numerically similar between vaccine types, at 8.7% (95% confidence interval −15.4% to −1.4%, P=0.02) for an adenovirus vector vaccine and 8.9% (−17.6% to 0.7%, P=0.07) for a mRNA vaccine.

**Table 3:** Estimated time trajectories of long covid from SARS-CoV-2 infection, and changes in trajectories after covid-19 vaccination, moderated by vaccine type

Outcomes	Estimate (SE)	P value	Odds ratio (95% CI)
**Long covid of any severity**			
Time trajectory (per week)	−0.004 (0.003)	0.19	0.996 (0.990 to 1.002)
First vaccine dose (change in level)	−0.093 (0.055)	0.09	0.911 (0.818 to 1.014)
Second vaccine dose (change in level)	−0.093 (0.051)	0.07	0.911 (0.824 to 1.007)
Time since first vaccination (per week)†	0.000 (0.008)	0.95	1.000 (0.985 to 1.016)
Time since second vaccination (per week)†	−0.004 (0.008)	0.65	0.996 (0.980 to 1.013)
Vaccine type: adenovirus vector (versus mRNA)	0.046 (0.055)	0.40	1.048 (0.941 to 1.166)
First vaccination interacted with type‡	−0.069 (0.067)	0.31	0.934 (0.818 to 1.066)
Second vaccination interacted with type‡	0.002 (0.064)	0.97	1.002 (0.883 to 1.137)
Time since first vaccination interacted with type§	0.009 (0.009)	0.33	1.009 (0.991 to 1.028)
Time since second vaccination interacted with type§	−0.010 (0.010)	0.33	0.990 (0.970 to 1.010)
**Activity-limiting long covid**			
Time trajectory (per week)	0.002 (0.004)	0.56	1.002 (0.995 to 1.009)
First vaccine dose (change in level)	−0.154 (0.070)	0.03	0.857 (0.747 to 0.984)
Second vaccine dose (change in level)	−0.026 (0.064)	0.68	0.974 (0.860 to 1.103)
Time since first vaccination (per week)†	0.003 (0.010)	0.77	1.003 (0.984 to 1.022)
Time since second vaccination (per week)†	−0.013 (0.010)	0.21	0.987 (0.968 to 1.007)
Vaccine type: adenovirus vector (versus mRNA)	0.042 (0.069)	0.54	1.043 (0.911 to 1.195)
First vaccination interacted with type‡	0.045 (0.087)	0.60	1.046 (0.883 to 1.240)
Second vaccination interacted with type‡	−0.116 (0.080)	0.15	0.890 (0.761 to 1.041)
Time since first vaccination interacted with type§	0.004 (0.012)	0.75	1.004 (0.981 to 1.027)
Time since second vaccination interacted with type§	0.004 (0.013)	0.73	1.004 (0.980 to 1.029)

CI=confidence interval; SE=standard error.

Estimates and odds ratios are adjusted for age, sex, white or non-white ethnicity, region or country, area deprivation fifth group, health status, patient-facing health or social care worker, hospital admission with acute covid-19, and calendar time of infection.

* Estimates and standard errors are on the logit scale.

† Odds ratios represent modification of the time trajectory.

‡ Odds ratios represent modification of the change in level after first or second vaccination by vaccine type.

§ Odds ratios represent modification of the time trajectory, modified by vaccine type.

The odds of long covid after a first dose of a covid-19 vaccine numerically decreased with duration from SARS-CoV-2 infection, with estimated numerical decreases of 24.8%, 16.5%, and 4.8% for participants who received a first vaccine dose 9, 12, and 15 months after infection (see supplementary figures 4a-b). Duration from infection to first vaccine dose, however, was not a statistically significant moderator of the vaccination-long covid relationship (see supplementary tables 5a-d).

We found no statistical evidence of differences in post-vaccination long covid trends according to sociodemographic characteristics (age, sex, ethnic group, area deprivation) or health related factors (self-reported health status not related to covid-19, whether admitted to hospital with acute covid-19) (see supplementary tables 5a-d).

### Trajectories of individual symptoms

**Fig 4:**
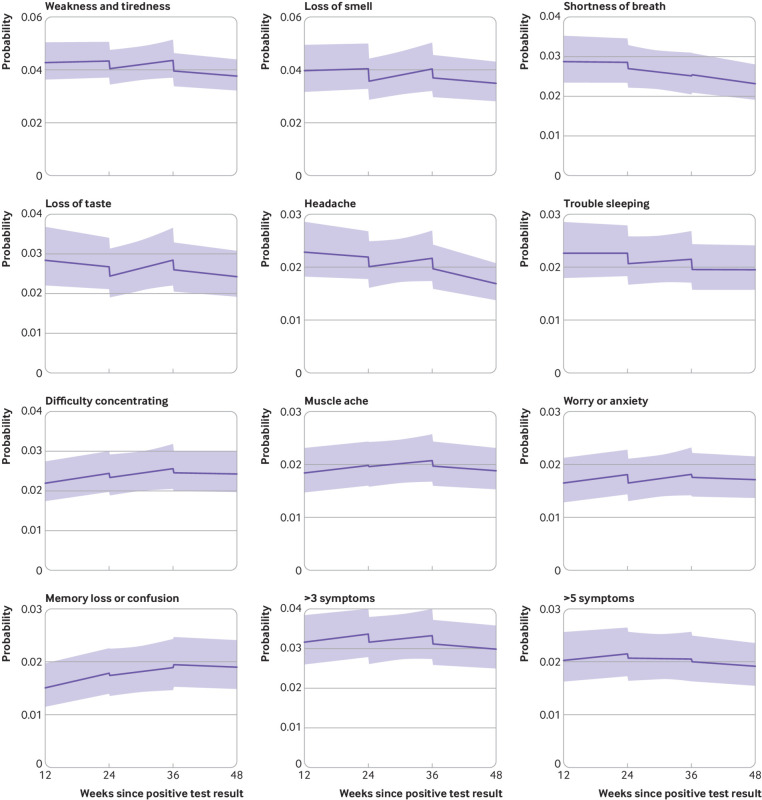
Modelled probabilities of individual long covid symptoms for a hypothetical study participant who received a first dose of a covid-19 vaccine 24 weeks after SARS-CoV-2 infection and a second dose 12 weeks later. Top 10 most frequently reported symptoms ordered by modelled probability at 12 weeks post-infection. Probabilities are shown for a participant of mean age (50 years) and in the modal group for other covariates (woman, white ethnicity, resident in London, resident in an area in the least deprived fifth group, not a patient-facing health or social care worker, no pre-existing health conditions, not admitted to hospital during the acute phase of infection, infected on 7 September 2020). Although the estimated probabilities are specific to this profile, proportional changes in probabilities after vaccination do not vary across characteristics and can therefore be generalised to other profiles. Dashed lines represent timing of vaccination. Shaded areas are 95% confidence intervals

The odds of experiencing most symptoms, as well as more than three or more than five symptoms together, initially numerically decreased after each vaccination (fig 4). After a first vaccine dose, the largest numerical decreases were observed for loss of smell (−12.5%, 95% confidence interval −21.5% to −2.5%, P=0.02), loss of taste (−9.2%, −19.8% to 2.7%, P=0.13), and trouble sleeping (−8.8%, −19.4% to 3.3%, P=0.15). After a second vaccine dose, the largest numerical decreases were observed for fatigue (−9.7%, −16.5% to −2.4%, P=0.01), headache (−9.0%, −18.1% to 1.0%, P=0.08), and trouble sleeping (−9.0%, −18.2% to 1.2%, P=0.08).

Similar to long covid overall, the odds of experiencing most individual symptoms and more than three or more than five symptoms together, numerically decreased after the first vaccine dose. Trends were generally upwards between the first and second vaccine doses, with most returning to a declining or flat trend after the second dose. However, owing to lack of statistical power, the data for most symptoms were compatible with both initial increases and decreases and both upward and downward trends in the likelihood of experiencing symptoms after each vaccine dose (see supplementary table 6).

## Discussion

In this community based study of adults aged 18 to 69 years infected with SARS-CoV-2 before vaccination against covid-19, we found that the odds of experiencing long covid symptoms that persisted for at least 12 weeks decreased by an average of 13% after a first covid-19 vaccine dose. It is, however, unclear from the data whether the improvement was sustained until a second vaccine dose was administered. Receiving a second vaccine dose was associated with a further 9% decrease in the odds of long covid, and statistical evidence suggested a sustained improvement after this, at least over the median follow-up of 67 days. Similar findings were obtained when the focus was on long covid severe enough to result in functional impairment.

We found no statistical evidence of heterogeneity in the associations between vaccination and long covid symptoms according to vaccine type; duration from infection to first vaccination; sociodemographic characteristics, such as age, sex, ethnicity, and area deprivation; self-reported health status; and hospital admission with acute covid-19. This observational study was unlikely to have been sufficiently powered to detect these associations, however, particularly given the multiplicity of testing, and absence of evidence does not necessarily imply evidence of absence.

### Findings in context

Our results add to existing evidence on the trajectory of long covid after vaccination. A non-controlled study of 900 social media users found that more than half had experienced an improvement in symptoms after vaccination compared with 7% who reported a deterioration.^19^ A study of 44 vaccinated patients and 22 unvaccinated controls previously admitted to hospital with covid-19 in the UK, which inevitably had limited power to detect clinically relevant effects, found no evidence for vaccination being associated with worsening of long covid symptoms or quality of life.^20^ A French study of 455 self-selected participants found reduced symptom burden and double the rate of remission at 120 days in vaccinated participants compared with unvaccinated controls.^21^

Vaccination against covid-19 effectively reduces rates of infection^12^ ^13^ and transmission.^14^ Evidence also suggests that the incidence of long covid is reduced in those infected after vaccination; in a study of 906 mobile phone app users, the odds of having symptoms ≥28 days post-infection was approximately halved in fully vaccinated participants compared with unvaccinated controls.^17^ Together with our results, these findings suggest that vaccination against covid-19 might reduce the population prevalence of long covid by reducing the risk of continuing to experience persistent symptoms in those who already have symptoms when vaccinated; developing persistent symptoms after breakthrough infections; being infected in the first place; and transmitting the virus after infection.

Our principal finding, of a decrease in the likelihood of experiencing long covid symptoms after a second covid-19 vaccine dose, supports hypothesised biological mechanisms. People with long covid who experience dysregulation of the immune system may benefit from autoimmune processes being “reset” by vaccination (although whether this is long lasting remains to be established), while any residual viral reservoir may also be destroyed by the antibody response.^34^ Immunological phenotyping suggests differences in those who experience persistent symptoms after SARS-CoV-2 infection compared with healthy controls.^35^ The presence of autoantibodies against interferon type I or autoimmune processes triggered by SARS-CoV-2 through molecular mimicry has been proposed as a manifestation of immune dysregulation in long covid, possibly similar to autoimmune rheumatic diseases.^36^ Another proposed mechanism is the persistence of viral antigen modifying the immune response months after infection.^37^ In this scenario, it is reasonable to hypothesise that covid-19 vaccination may be beneficial.

The symptom trajectory following the initial decrease after a first vaccine dose was unclear, being compatible with both increasing and decreasing odds of long covid over time. Evidence was, however, found of an increasing trend when the sample was restricted to participants vaccinated during the follow-up period. Relapsing symptoms are common in long covid,^4^ ^5^ ^6^ and persistent symptoms are associated with a weak antibody response,^38^ so it is possible that receiving a first vaccine dose alone is insufficient for sustained improvement in some people.

UK Government guidelines recommend that people should delay vaccination for four weeks after a positive test result for SARS-CoV-2. Given that we only considered follow-up visits beyond 12 weeks of participants’ first positive test result (as our outcome of interest was long covid symptoms at least 12 weeks after infection), it seems unlikely that this guidance would have affected our analysis. The National Health Service advises patients with ongoing complications of covid-19 to consult their doctor about vaccination, so it is possible that some people with long covid symptoms may choose to delay vaccination until their symptoms resolve, which would induce an association. However, this is unlikely to have affected our analysis, as we found no evidence that study participants with long covid deferred their covid-19 vaccination compared with those without long covid (see supplementary figure 5). Because our study was observational, we cannot rule out the possibility of a change in reported symptoms after vaccination being due to a placebo effect. Although we might expect to observe a post-vaccination relapse in some participants whose symptoms initially improved, our ability to do this was limited by the follow-up available to us (a median of 67 days from the second vaccine dose).

### Strengths and limitations of this study

The main strength of the study is its use of the ONS COVID-19 Infection Survey, a large survey of about half a million people from the community dwelling population of the UK with longitudinal follow-up. Random sampling from address lists mitigates against selection bias, whereas the prospective design means that survey responses are not subject to outcome recall bias (such as participants overestimating the duration of previously experienced symptoms). All of the survey’s participants are swabbed for SARS-CoV-2 at every follow-up visit, irrespective of symptoms, so our study includes asymptomatic as well symptomatic infections.

The study also has limitations. Its observational design means that causality cannot be inferred, and placebo and side effects of vaccination may have contributed to our findings; however, estimates were robust to excluding follow-up visits within the first week of each vaccination, suggesting that the impact of these effects is likely to be small. Although we adjusted for a wide range of potential confounders, unmeasured factors, such as those related to take-up of a second vaccine dose, may remain. The observed changes after vaccination could be related to the relapsing and remitting symptoms experienced by many people living with long covid^4^ ^5^ ^6^ rather than to a causal effect of the vaccine. Future analysis should consider differing patterns of illness, including quantification of the frequency and duration of symptom-free periods after vaccination.

By definition, symptoms are self-reported with no other way to assess them. Together with the impact of symptoms on daily life, the presence of symptoms is how WHO defines long covid.^25^ However, attributing symptoms to a previous SARS-CoV-2 infection is likely to be more difficult in the absence of a diagnostic test for long covid and probably results in under-recording in electronic health records.^39^ Although all infections in this study were confirmed by testing, long covid status was self-reported and we did not have data on related healthcare use, so we cannot exclude some participants’ symptoms being caused by a medical condition other than covid-19. Although clinical case definitions for long covid exist in the UK^2^ and internationally,^25^ consensus over a suitable working definition for research purposes is lacking; thus there is potential for inconsistencies in outcome measurement between our study and other studies on long covid. Based on the same data source as that used in this study, ONS previously estimated 12 week prevalence rates from 3% (based on tracking 12 specific symptoms) to 12% (based on self-classification of long covid),^40^ showing the sensitivity of estimates as to how long covid prevalence is measured. Nonetheless, we investigated changes in the illness trajectory after vaccination rather than estimate the prevalence of long covid at a particular time point, and our outcome definition was consistent over the study period.

Given the staged roll-out of the vaccination programme in the UK, the main determinant of vaccination timing is age, and thus older study participants tended to have longer post-vaccination follow-up time than younger participants; this may have influenced our analysis of effect modification, whereby we found no evidence of heterogeneity in the post-vaccination trajectory of long covid according to age group. The measure of functional impairment recorded on the COVID-19 Infection Survey (day-to-day activities: not limited, limited a little, or limited a lot) did not give a detailed indication of the specific ways that participants’ lives have been affected by long covid or the resulting impact on quality of life. It is possible that the average improvement in long covid symptoms and functional impact may wane with time, and longer term follow-up is required to establish whether the estimated changes after second vaccination are sustained. Follow-up after a booster dose—now widely available in the UK adult population—is also required. The study sample was restricted to participants aged 18 to 69 years, so our findings may not generalise to children or older adults, nor may they apply to people who had not received a vaccine by 5 September 2021, in particular those with vaccine hesitancy because of their long covid symptoms. Furthermore, symptom data were collected prospectively rather than retrospectively, so variable estimates relating to changes in the odds of long covid may not be generalisable to participants who were vaccinated before the long covid question was added to the COVID-19 Infection Survey on 3 February 2021. However, our results were insensitive to inclusion of participants who remained unvaccinated by the end of the study period or to exclusion of those who were vaccinated before the start of the study period.

### Conclusions

We found that vaccination against covid-19 is associated with a decrease in the likelihood of continuing to experience long covid symptoms in adults aged 18 to 69 years, and this appeared to be sustained after a second vaccine dose. Our results suggest that vaccination of people previously infected may be associated with a reduction in the burden of long covid on population health, at least in the first few months after vaccination. Further research is required to evaluate the long term relationship between vaccination and long covid, in particular the impact of the omicron variant, which has become dominant in the UK, booster doses, now widely available to adults in the population, and reinfections. Studies are also needed to understand the biological mechanisms underpinning any improvements in symptoms after vaccination, which may contribute to the development of therapeutics for long covid.

**What is already known on this topic**

Vaccines against covid-19 are effective at reducing rates of SARS-CoV-2 infection, transmission, hospital admission, and death
The incidence of long covid may be reduced in those who are infected after vaccination, but the relationship between vaccination and pre-existing long covid symptoms is unclear, as published studies are generally small and with self-selected participants

**What this study adds**

A first dose of covid-19 vaccine was associated with a reduction in long covid symptoms, and evidence suggested a sustained improvement after a second dose, at least over the median follow-up of 67 days in this study
No evidence was found of differences in this relationship by sociodemographic characteristics, health related factors, vaccine type, or duration from infection to vaccination
Although causality cannot be inferred from this observational evidence, vaccination may contribute to a reduction in the population health burden of long covid

## Data Availability

Deidentified study data are available to accredited researchers in the ONS Secure Research Service under part 5, chapter 5 of the Digital Economy Act 2017. For further information about accreditation, contact research.support@ons.gov.uk or visit ons.gov.uk/aboutus/whatwedo/statistics/requestingstatistics/approvedresearcherscheme.

## Web extra

Extra material supplied by authors

## Notes

Contributors: DA, KBP, MG, VN, NAA, and ASW conceptualised and designed the study. DA and CB prepared the study data and performed the statistical analysis. All authors contributed to interpretation of the results. DA and CB were responsible for the first draft of the manuscript. All authors contributed to critical revision of the manuscript. All authors approved the final manuscript. DA is the guarantor. The corresponding author attests that all listed authors meet authorship criteria and that no others meeting the criteria have been omitted.

The lead author (DA) affirms that the manuscript is an honest, accurate, and transparent account of the study being reported; that no important aspects of the study have been omitted; and that any discrepancies from the study as planned (and, if relevant, registered) have been explained.

Dissemination to participants and related patient and public communities: The use of deidentified data precludes direct dissemination to participants. For the purpose of open access, the authors have applied a Creative Commons Attribution (CC BY) licence to any Author Accepted Manuscript version arising.

Provenance and peer review: Not commissioned; externally peer reviewed.

## References

[1] UK government. Coronavirus (COVID-19) in the UK. 18 February 2022. https://coronavirus.data.gov.uk

[2] National Institute for Health and Care Excellence. COVID-19 rapid guideline: managing the long-term effects of COVID-19. 2021. www.nice.org.uk/guidance/ng188 33555768

[3] Office for National Statistics. Prevalence of ongoing symptoms following coronavirus (COVID-19) infection in the UK: 2 December 2021. 2021. www.ons.gov.uk/peoplepopulationandcommunity/healthandsocialcare/conditionsanddiseases/bulletins/prevalenceofongoingsymptomsfollowingcoronaviruscovid19infectionintheuk/2december2021

[4] National Institute for Health Research. Living with Covid19 – Second review. 2021. https://evidence.nihr.ac.uk/themedreview/living-with-covid19-second-review

[5] Ziauddeen N Gurdasani D O’Hara ME . Characteristics of Long Covid: findings from a social media survey. medRxiv 2021. www.medrxiv.org/content/10.1101/2021.03.21.21253968v2 10.1136/jech-2021-SSMabstracts.194

[6] Davis HE Assaf GS McCorkell L . Characterizing long COVID in an international cohort: 7 months of symptoms and their impact. EClinicalMedicine 2021;38:101019. 10.1016/j.eclinm.2021.101019. 34308300 PMC8280690

[7] Whitaker M Elliott J Chadeau-Hyam M . Persistent symptoms following SARS-CoV-2 infection in a random community sample of 508 707 people. medRxiv 2021. https://www.medrxiv.org/content/10.1101/2021.06.28.21259452v1

[8] Falsey AR Sobieszczyk ME Hirsch I AstraZeneca AZD1222 Clinical Study Group . Phase 3 Safety and Efficacy of AZD1222 (ChAdOx1 nCoV-19) Covid-19 Vaccine. N Engl J Med 2021;385:2348-60. 10.1056/NEJMoa2105290. 34587382 PMC8522798

[9] Voysey M Clemens SAC Madhi SA Oxford COVID Vaccine Trial Group . Safety and efficacy of the ChAdOx1 nCoV-19 vaccine (AZD1222) against SARS-CoV-2: an interim analysis of four randomised controlled trials in Brazil, South Africa, and the UK. Lancet 2021;397:99-111. 10.1016/S0140-6736(20)32661-1. 33306989 PMC7723445

[10] Polack FP Thomas SJ Kitchin N C4591001 Clinical Trial Group . Safety and Efficacy of the BNT162b2 mRNA Covid-19 Vaccine. N Engl J Med 2020;383:2603-15. 10.1056/NEJMoa2034577. 33301246 PMC7745181

[11] Baden LR El Sahly HM Essink B COVE Study Group . Efficacy and Safety of the mRNA-1273 SARS-CoV-2 Vaccine. N Engl J Med 2021;384:403-16. 10.1056/NEJMoa2035389. 33378609 PMC7787219

[12] Pritchard E Matthews PC Stoesser N . Impact of vaccination on new SARS-CoV-2 infections in the United Kingdom. Nat Med 2021;27:1370-8. 10.1038/s41591-021-01410-w. 34108716 PMC8363500

[13] Menni C Klaser K May A . Vaccine side-effects and SARS-CoV-2 infection after vaccination in users of the COVID Symptom Study app in the UK: a prospective observational study. Lancet Infect Dis 2021;21:939-49. 10.1016/S1473-3099(21)00224-3. 33930320 PMC8078878

[14] Harris RJ Hall JA Zaidi A Andrews NJ Dunbar JK Dabrera G . Effect of Vaccination on Household Transmission of SARS-CoV-2 in England. N Engl J Med 2021;385:759-60. 10.1056/NEJMc2107717. 34161702 PMC8262621

[15] Lopez Bernal J Andrews N Gower C . Effectiveness of the Pfizer-BioNTech and Oxford-AstraZeneca vaccines on covid-19 related symptoms, hospital admissions, and mortality in older adults in England: test negative case-control study. BMJ 2021;373:n1088. 10.1136/bmj.n1088. 33985964 PMC8116636

[16] Office for National Statistics. Deaths involving COVID-19 by vaccination status, England: deaths occurring between 2 January and 24 September 2021. 2021. www.ons.gov.uk/peoplepopulationandcommunity/birthsdeathsandmarriages/deaths/bulletins/deathsinvolvingcovid19byvaccinationstatusengland/deathsoccurringbetween2januaryand24september2021

[17] Antonelli M Penfold RS Merino J . Risk factors and disease profile of post-vaccination SARS-CoV-2 infection in UK users of the COVID Symptom Study app: a prospective, community-based, nested, case-control study. Lancet Infect Dis 2022;22:43-55. 10.1016/S1473-3099(21)00460-6. 34480857 PMC8409907

[18] Massey D Berrent D Akrami A . Change in Symptoms and Immune Response in People with Post-Acute Sequelae of SARS-Cov-2 Infection (PASC) After SARS-Cov-2 Vaccination. medRxiv 2021. www.medrxiv.org/content/10.1101/2021.07.21.21260391v2 10.1101/2021.07.21.21260391

[19] Sherwood O, Strain D, Rossman J. The impact of COVID vaccination on symptoms of Long Covid. An international survey of 900 people with lived experience. 2021. https://3ca26cd7-266e-4609-b25f-6f3d1497c4cf.filesusr.com/ugd/8bd4fe_a338597f76bf4279a851a7a4cb0e0a74.pdf 10.3390/vaccines10050652 PMC9146071 35632408

[20] Arnold DT Milne A Samms E Stadon L Maskell NA Hamilton FW . Are vaccines safe in patients with Long COVID? A prospective observational study. medRxiv 2021. www.medrxiv.org/content/10.1101/2021.03.11.21253225v3 10.1101/2021.03.11.21253225

[21] Tran V-T Perrodeau E Saldanha J Pane I Ravaud P . Efficacy of COVID-19 Vaccination on the Symptoms of Patients With Long COVID: A Target Trial Emulation Using Data From the ComPaRe e-Cohort in France. SSRN 2021. https://papers.ssrn.com/sol3/papers.cfm?abstract_id=3932953 10.1136/bmjmed-2022-000229 PMC9978748 36910458

[22] Miyake E Martin S . Long Covid: Online patient narratives, public health communication and vaccine hesitancy. Digit Health 2021;7:20552076211059649. 10.1177/20552076211059649. 34868622 PMC8638072

[23] Office for National Statistics. COVID-19 Infection Survey: methods and further information. 2021. www.ons.gov.uk/peoplepopulationandcommunity/healthandsocialcare/conditionsanddiseases/methodologies/covid19infectionsurveypilotmethodsandfurtherinformation

[24] Michelen M Manoharan L Elkheir N . Characterising long COVID: a living systematic review. BMJ Glob Health 2021;6:e005427. 10.1136/bmjgh-2021-005427. 34580069 PMC8478580

[25] World Health Organisation. A clinical case definition of post COVID-19 condition by a Delphi consensus, 6 October 2021. 2021. www.who.int/publications-detail-redirect/WHO-2019-nCoV-Post_COVID-19_condition-Clinical_case_definition-2021.1

[26] UK Government. COVID-19: the green book, chapter 14a. 2020. www.gov.uk/government/publications/covid-19-the-green-book-chapter-14a

[27] Public Health England. COVID-19: the green book, chapter 14a. 2020. www.gov.uk/government/publications/covid-19-the-green-book-chapter-14a

[28] Austin PC . Balance diagnostics for comparing the distribution of baseline covariates between treatment groups in propensity-score matched samples. Stat Med 2009;28:3083-107. 10.1002/sim.3697. 19757444 PMC3472075

[29] Bernal JL Cummins S Gasparrini A . Interrupted time series regression for the evaluation of public health interventions: a tutorial. Int J Epidemiol 2017;46:348-55. 10.1093/ije/dyw098. 27283160 PMC5407170

[30] Office for National Statistics. Coronavirus (COVID-19) Infection Survey technical article: waves and lags of COVID-19 in England, June 2021. 2021. www.ons.gov.uk/peoplepopulationandcommunity/healthandsocialcare/conditionsanddiseases/articles/coronaviruscovid19infectionsurveytechnicalarticle/wavesandlagsofcovid19inenglandjune2021

[31] Centres for Disease Control and Prevention. Symptoms of COVID-19. 2021. www.cdc.gov/coronavirus/2019-ncov/symptoms-testing/symptoms.html

[32] Alwan NA . Lessons from Long COVID: working with patients to design better research. Nat Rev Immunol 2022;22:201-2. 10.1038/s41577-022-00692-6. 35169259 PMC8853146

[33] Alwan NA . The teachings of Long COVID. Commun Med 2021;1:15. 10.1038/s43856-021-00016-0. PMC9053272 35602198

[34] Massey D Berrent D Akrami A . Change in Symptoms and Immune Response in People with Post-Acute Sequelae of SARS-Cov-2 Infection (PASC) After SARS-Cov-2 Vaccination. medRxiv 2021. www.medrxiv.org/content/10.1101/2021.07.21.21260391v2 10.1101/2021.07.21.21260391

[35] Ryan FJ Hope CM Masavuli MG . Long-term perturbation of the peripheral immune system months after SARS-CoV-2 infection. BMC Med 2022;20:26. 10.1186/s12916-021-02228-6. 35027067 PMC8758383

[36] Drosos AA Pelechas E Voulgari PV . Long COVID from rheumatology perspective: a simple mimicker or promoter of autoimmunity? Clin Rheumatol 2022;41:957-8. 10.1007/s10067-022-06092-4. 35147823 PMC8831874

[37] Gaebler C Wang Z Lorenzi JCC . Evolution of antibody immunity to SARS-CoV-2. Nature 2021;591:639-44. 10.1038/s41586-021-03207-w. 33461210 PMC8221082

[38] García-Abellán J Padilla S Fernández-González M . Antibody Response to SARS-CoV-2 is Associated with Long-term Clinical Outcome in Patients with COVID-19: a Longitudinal Study. J Clin Immunol 2021;41:1490-501. 10.1007/s10875-021-01083-7. 34273064 PMC8285689

[39] Walker AJ MacKenna B Inglesby P The OpenSAFELY Collaborative ). Clinical coding of long COVID in English primary care: a federated analysis of 58 million patient records *in situ* using OpenSAFELY. Br J Gen Pract 2021;71:e806-14. 10.3399/BJGP.2021.0301. 34340970 PMC8340730

[40] Office for National Statistics. Technical article: Updated estimates of the prevalence of post-acute symptoms among people with coronavirus (COVID-19) in the UK: 26 April 2020 to 1 August 2021. 2021. www.ons.gov.uk/peoplepopulationandcommunity/healthandsocialcare/conditionsanddiseases/articles/technicalarticleupdatedestimatesoftheprevalenceofpostacutesymptomsamongpeoplewithcoronaviruscovid19intheuk/26april2020to1august2021

