# Supplementary material for: Trajectory of long covid symptoms after covid-19 vaccination: community based cohort study
Source: BMJ. 2022 May 18;377:e069676. doi: 10.1136/bmj-2021-069676 (PMC9115603; doi:10.1136/bmj-2021-069676)
Supplement: Supplementary file 1 — Supplementary information: additional tables 1-6, figures 1-5 [file ayod069676.ww.pdf]

Supplementary Table 1. Number and percentage of households invited to participate in the Coronavirus (COVID-19) Infection Survey who subsequently enrolled, by country and phase

Phase	England	Wales	Northern Ireland	Scotland
Initial invitation	10,266 (51%)	7,017 (40%)	7,084 (43%)	N/A
Extension period	39,345 (43%)	N/A	N/A	N/A
AddressBase	167,890 (12%)	7,013 (14%)	N/A	22,131 (13%)

Notes: The initial invitation phase was open to previous respondents to ONS surveys who had consented to participate in future research, and started on 26 April 2020 in England, 29 June 2020 in Wales, and 26 July 2020 in Northern Ireland. The extension period refers to the period of time beyond the initial pilot phase of the study when the sample was increased, and started on 31 May 2020 in England. Sampling from AddressBase started on 13 July 2020 in England, 5 October 2020 in Wales, and 14 September 2020 in Scotland, and involves randomly selecting addresses from an address list. Enrolment rates as of 22 October 2021, taken from the technical dataset accompanying the Coronavirus (COVID-19) Infection Survey: https://www.ons.gov.uk/peoplepopulationandcommunity/healthandsocialcare/conditionsanddiseases/datasets/covid19infectionsurveytechnicaldata

Supplementary Table 2. Long Covid symptoms included on the Coronavirus (COVID-19) Infection Survey

**Symptom**
1. Fever
2. Headache
3. Muscle ache
4. Weakness/tiredness
5. Nausea/vomiting
6. Abdominal pain
7. Diarrhoea
8. Loss of appetite
9. Loss of taste
10. Loss of smell
11. Sore throat
12. Cough
13. Shortness of breath
14. Chest pain
15. Palpitations
16. Vertigo/dizziness
17. Worry/anxiety
18. Low mood/not enjoying anything
19. Trouble sleeping
20. Memory loss or confusion
21. Difficulty concentrating

Notes: All participants who responded positively to the survey question “Would you describe yourself as having 'long COVID', that is, you are still experiencing symptoms more than 4 weeks after you first had COVID-19, that are not explained by something else?” were then asked “Do you have any of the following symptoms as part of your experience of long COVID? Please include any pre-existing symptoms which long COVID has made worse (answer Yes or No for each one).”

Supplementary Table 3. Description of covariates included in the analysis

Covariate	Specification in analysis
Time since infection date (days)	Linear
Calendar time of infection (days)	Restricted cubic spline with boundary knots at the 10th and 90th percentiles and an interior knot at the 50th percentile
Age (years)	Restricted cubic spline with boundary knots at the 10th and 90th percentiles and an interior knot at the 50th percentile
Sex	Binary dummy variable (male [reference], female)
Ethnic group	Binary dummy variable (white [reference], non-white)
Region or country	Categorical variable (North East England, North West England, Yorkshire and the Humber, East Midlands, West Midlands, East of England, London [reference], South East England, South West England, Northern Ireland, Scotland, Wales)
Area deprivation quintile group	Categorical variable (1, most deprived [reference], to 5, least deprived)
Patient-facing health or social care worker	Binary dummy variable (no [reference], yes)
Health conditions (excluding those attributed to COVID-19)	Binary dummy variable (no [reference], yes)
Hospitalisation with acute COVID-19	Binary dummy variable (no [reference], yes)

Notes: Calendar time of infection was calculated as the number of days from 24 January 2020 (when the first SARS-CoV-2 cases were reported in the UK) and the infection date. The study sample size did not permit disaggregation of ethnicity beyond white and non-white groups. Area deprivation was based on the English Indices of Deprivation 2019, the Welsh Index of Multiple Deprivation 2019, the Scottish Index of Multiple Deprivation 2020, and the Northern Ireland Multiple Deprivation Measure 2017. Health conditions were self-reported rather than clinically diagnosed based on the survey question: "Do you have any physical or mental health conditions or illnesses lasting or expected to last 12 months or more (excluding any long-lasting COVID-19 symptoms)?" Hospitalisation with acute COVID-19 was self-reported rather than derived from medical records.

Supplementary Table 4. Number and percentage of included participants who received their first and second vaccinations during (rather than before) the follow-up period, stratified by age group and health conditions at last follow-up visit

Age group	Health conditions	Total	Received first vaccination during follow-up	Received second vaccination during follow-up
18 to 29 years	No	3,789	2,367 (62.5%)	1,666 (44.0%)
	Yes	385	197 (51.2%)	213 (55.3%)
30 to 39 years	No	4,902	2,921 (59.6%)	3,264 (66.6%)
	Yes	533	238 (44.7%)	359 (67.4%)
40 to 49 years	No	5,675	2,923 (51.5%)	4,325 (76.2%)
	Yes	699	291 (41.6%)	534 (76.4%)
50 to 59 years	No	5,829	2,275 (39.0%)	4,719 (81.0%)
	Yes	1,112	394 (35.4%)	888 (79.9%)
60 to 70 years	No	4,310	1,088 (25.2%)	3,507 (81.4%)
	Yes	1,122	277 (24.7%)	860 (76.7%)

Notes: The study sample included only people aged under 70 years at their first visit during the follow-up period, and a small number of participants turned 70 before their last visit, hence the upper boundary of the final age group is 70 rather than 69 years. Health conditions were self-reported rather than clinically diagnosed based on the survey question: "Do you have any physical or mental health conditions or illnesses lasting or expected to last 12 months or more (excluding any long-lasting COVID-19 symptoms)?"

Supplementary Table 5a. Interactions between vaccination and personal characteristics: Long Covid of any severity, first vaccination

Modifier (comparator)	Exposure	Group	Estimate	SE	P-value	H-B p-value	B-Y p-value
Vaccine type (mRNA vaccine)	First vaccination (change in level)	Adenovirus vector	-0.069	0.067	0.31	>0.99	>0.99
	Time since first vaccination (weeks)	Adenovirus vector	0.009	0.009	0.33	>0.99	>0.99
Age group (18 to 29 years)	First vaccination (change in level)	30 to 39 years	0.157	0.132	0.23	>0.99	>0.99
		40 to 49 years	0.195	0.129	0.13	>0.99	>0.99
		50 to 59 years	0.136	0.131	0.30	>0.99	>0.99
		60 to 70 years	-0.021	0.143	0.89	>0.99	>0.99
	Time since first vaccination (weeks)	30 to 39 years	-0.028	0.021	0.18	>0.99	>0.99
		40 to 49 years	-0.024	0.020	0.24	>0.99	>0.99
		50 to 59 years	-0.029	0.020	0.15	>0.99	>0.99
		60 to 70 years	-0.024	0.020	0.24	>0.99	>0.99
Sex (male)	First vaccination (change in level)	Female	-0.044	0.068	0.52	>0.99	>0.99
	Time since first vaccination (weeks)	Female	0.016	0.009	0.08	>0.99	>0.99
Ethnic group (white)	First vaccination (change in level)	Non-white	0.059	0.121	0.62	>0.99	>0.99
	Time since first vaccination (weeks)	Non-white	0.005	0.016	0.78	>0.99	>0.99
Area deprivation quintile group (1, most deprived)	First vaccination (change in level)	2	0.098	0.115	0.40	>0.99	>0.99
		3	-0.123	0.109	0.26	>0.99	>0.99
		4	-0.102	0.109	0.35	>0.99	>0.99
		5 (least deprived)	0.064	0.108	0.55	>0.99	>0.99
	Time since first vaccination (weeks)	2	-0.012	0.015	0.40	>0.99	>0.99
		3	0.012	0.014	0.40	>0.99	>0.99
		4	0.006	0.014	0.65	>0.99	>0.99
		5 (least deprived)	-0.029	0.014	0.046	>0.99	>0.99
Health conditions (No)	First vaccination (change in level)	Yes	-0.015	0.080	0.85	>0.99	>0.99
	Time since first vaccination (weeks)	Yes	0.007	0.009	0.45	>0.99	>0.99
Hospitalised at acute phase of infection (No)	First vaccination (change in level)	Yes	0.035	0.141	0.80	>0.99	>0.99
	Time since first vaccination (weeks)	Yes	0.005	0.015	0.76	>0.99	>0.99
Days from infection to vaccination (restricted cubic spline)	First vaccination (change in level)	First term	-0.527	0.299	0.08	>0.99	>0.99
		Second term	0.013	0.087	0.88	>0.99	>0.99
	Time since first vaccination (weeks)	First term	0.051	0.023	0.03	>0.99	>0.99
		Second term	0.016	0.011	0.15	>0.99	>0.99

Notes: B-Y: Benjamini-Yekutieli; H-B: Holm-Bonferroni; mRNA: messenger ribonucleic acid; SE: standard error.

Supplementary Table 5b. Interactions between vaccination and personal characteristics: Long Covid of any severity, second vaccination

Modifier (comparator)	Exposure	Group	Estimate	SE	P-value	H-B p-value	B-Y p-value
Vaccine type (mRNA vaccine)	Second vaccination (change in level)	Adenovirus vector	0.002	0.064	0.97	>0.99	>0.99
	Time since second vaccination (weeks)	Adenovirus vector	-0.010	0.010	0.33	>0.99	>0.99
Age group (18 to 29 years)	Second vaccination (change in level)	30 to 39 years	0.182	0.145	0.21	>0.99	>0.99
		40 to 49 years	0.206	0.136	0.13	>0.99	>0.99
		50 to 59 years	0.239	0.134	0.07	>0.99	>0.99
		60 to 70 years	0.177	0.137	0.19	>0.99	>0.99
	Time since second vaccination (weeks)	30 to 39 years	0.041	0.026	0.12	>0.99	>0.99
		40 to 49 years	0.037	0.025	0.15	>0.99	>0.99
		50 to 59 years	0.035	0.025	0.17	>0.99	>0.99
		60 to 70 years	0.033	0.026	0.20	>0.99	>0.99
Sex (male)	Second vaccination (change in level)	Female	-0.124	0.064	0.06	>0.99	>0.99
	Time since second vaccination (weeks)	Female	-0.015	0.011	0.16	>0.99	>0.99
Ethnic group (white)	Second vaccination (change in level)	Non-white	-0.092	0.112	0.41	>0.99	>0.99
	Time since second vaccination (weeks)	Non-white	-0.004	0.018	0.81	>0.99	>0.99
Area deprivation quintile group (1, most deprived)	Second vaccination (change in level)	2	0.123	0.108	0.26	>0.99	>0.99
		3	0.026	0.105	0.81	>0.99	>0.99
		4	0.041	0.103	0.69	>0.99	>0.99
		5 (least deprived)	0.242	0.104	0.02	>0.99	>0.99
	Time since second vaccination (weeks)	2	0.001	0.017	0.94	>0.99	>0.99
		3	-0.026	0.016	0.09	>0.99	>0.99
		4	-0.015	0.015	0.34	>0.99	>0.99
		5 (least deprived)	0.017	0.016	0.30	>0.99	>0.99
Health conditions (No)	Second vaccination (change in level)	Yes	-0.049	0.071	0.49	>0.99	>0.99
	Time since second vaccination (weeks)	Yes	0.004	0.010	0.67	>0.99	>0.99
Hospitalised at acute phase of infection (No)	Second vaccination (change in level)	Yes	0.076	0.113	0.50	>0.99	>0.99
	Time since second vaccination (weeks)	Yes	-0.005	0.017	0.79	>0.99	>0.99
Days from infection to vaccination (restricted cubic spline)	Second vaccination (change in level)	First term	-0.107	0.142	0.45	>0.99	>0.99
		Second term	-0.025	0.085	0.77	>0.99	>0.99
	Time since second vaccination (weeks)	First term	-0.057	0.025	0.02	>0.99	>0.99
		Second term	-0.005	0.014	0.72	>0.99	>0.99

Notes: B-Y: Benjamini-Yekutieli; H-B: Holm-Bonferroni; mRNA: messenger ribonucleic acid; SE: standard error.

Supplementary Table 5c. Interactions between vaccination and personal characteristics: activity-limiting Long Covid, first vaccination

Modifier (comparator)	Exposure	Group	Estimate	SE	P-value	H-B p-value	B-Y p-value
Vaccine type (mRNA vaccine)	First vaccination (change in level)	Adenovirus vector	0.045	0.087	0.60	>0.99	>0.99
	Time since first vaccination (weeks)	Adenovirus vector	0.004	0.012	0.75	>0.99	>0.99
Age group (18 to 29 years)	First vaccination (change in level)	30 to 39 years	0.267	0.199	0.18	>0.99	>0.99
		40 to 49 years	0.306	0.195	0.12	>0.99	>0.99
		50 to 59 years	0.273	0.195	0.16	>0.99	>0.99
		60 to 70 years	0.203	0.206	0.33	>0.99	>0.99
	Time since first vaccination (weeks)	30 to 39 years	-0.029	0.032	0.36	>0.99	>0.99
		40 to 49 years	-0.024	0.031	0.45	>0.99	>0.99
		50 to 59 years	-0.025	0.031	0.42	>0.99	>0.99
		60 to 70 years	-0.023	0.031	0.45	>0.99	>0.99
Sex (male)	First vaccination (change in level)	Female	-0.123	0.086	0.15	>0.99	>0.99
	Time since first vaccination (weeks)	Female	0.023	0.011	0.04	>0.99	>0.99
Ethnic group (white)	First vaccination (change in level)	Non-white	-0.030	0.154	0.85	>0.99	>0.99
	Time since first vaccination (weeks)	Non-white	0.019	0.019	0.34	>0.99	>0.99
Area deprivation quintile group (1, most deprived)	First vaccination (change in level)	2	0.385	0.141	0.006	0.35	0.56
		3	0.045	0.133	0.74	>0.99	>0.99
		4	0.138	0.132	0.30	>0.99	>0.99
		5 (least deprived)	0.135	0.135	0.32	>0.99	>0.99
	Time since first vaccination (weeks)	2	-0.028	0.018	0.11	>0.99	>0.99
		3	0.008	0.016	0.62	>0.99	>0.99
		4	-0.015	0.016	0.36	>0.99	>0.99
		5 (least deprived)	-0.022	0.017	0.20	>0.99	>0.99
Health conditions (No)	First vaccination (change in level)	Yes	-0.030	0.092	0.75	>0.99	>0.99
	Time since first vaccination (weeks)	Yes	-0.001	0.010	0.91	>0.99	>0.99
Hospitalised at acute phase of infection (No)	First vaccination (change in level)	Yes	0.135	0.155	0.38	>0.99	>0.99
	Time since first vaccination (weeks)	Yes	-0.015	0.017	0.38	>0.99	>0.99
Days from infection to vaccination (restricted cubic spline)	First vaccination (change in level)	First term	-0.606	0.374	0.11	>0.99	>0.99
		Second term	-0.031	0.111	0.78	>0.99	>0.99
	Time since first vaccination (weeks)	First term	0.078	0.028	0.005	0.30	0.56
		Second term	0.027	0.013	0.04	>0.99	>0.99

Notes: B-Y: Benjamini-Yekutieli; H-B: Holm-Bonferroni; mRNA: messenger ribonucleic acid; SE: standard error.

Supplementary Table 5d. Interactions between vaccination and personal characteristics: activity-limiting Long Covid, second vaccination

Modifier (comparator)	Exposure	Group	Estimate	SE	P-value	H-B p-value	B-Y p-value
Vaccine type (mRNA vaccine)	Second vaccination (change in level)	Adenovirus vector	-0.116	0.080	0.15	>0.99	>0.99
	Time since second vaccination (weeks)	Adenovirus vector	0.004	0.013	0.73	>0.99	>0.99
Age group (18 to 29 years)	Second vaccination (change in level)	30 to 39 years	0.112	0.198	0.57	>0.99	>0.99
		40 to 49 years	0.051	0.187	0.79	>0.99	>0.99
		50 to 59 years	0.104	0.183	0.57	>0.99	>0.99
		60 to 70 years	0.065	0.185	0.73	>0.99	>0.99
	Time since second vaccination (weeks)	30 to 39 years	0.045	0.041	0.28	>0.99	>0.99
		40 to 49 years	0.035	0.040	0.38	>0.99	>0.99
		50 to 59 years	0.029	0.040	0.47	>0.99	>0.99
		60 to 70 years	0.027	0.040	0.50	>0.99	>0.99
Sex (male)	Second vaccination (change in level)	Female	-0.141	0.080	0.08	>0.99	>0.99
	Time since second vaccination (weeks)	Female	-0.025	0.013	0.047	>0.99	>0.99
Ethnic group (white)	Second vaccination (change in level)	Non-white	-0.169	0.138	0.22	>0.99	>0.99
	Time since second vaccination (weeks)	Non-white	-0.011	0.021	0.61	>0.99	>0.99
Area deprivation quintile group (1, most deprived)	Second vaccination (change in level)	2	0.190	0.129	0.14	>0.99	>0.99
		3	-0.046	0.125	0.71	>0.99	>0.99
		4	0.107	0.124	0.39	>0.99	>0.99
		5 (least deprived)	0.247	0.125	0.048	>0.99	>0.99
	Time since second vaccination (weeks)	2	0.017	0.020	0.40	>0.99	>0.99
		3	-0.022	0.018	0.22	>0.99	>0.99
		4	0.004	0.018	0.82	>0.99	>0.99
		5 (least deprived)	0.010	0.019	0.60	>0.99	>0.99
Health conditions (No)	Second vaccination (change in level)	Yes	0.031	0.080	0.70	>0.99	>0.99
	Time since second vaccination (weeks)	Yes	0.008	0.011	0.48	>0.99	>0.99
Hospitalised at acute phase of infection (No)	Second vaccination (change in level)	Yes	0.087	0.124	0.48	>0.99	>0.99
	Time since second vaccination (weeks)	Yes	0.014	0.019	0.45	>0.99	>0.99
Days from infection to vaccination (restricted cubic spline)	Second vaccination (change in level)	First term	-0.074	0.174	0.67	>0.99	>0.99
		Second term	-0.080	0.105	0.44	>0.99	>0.99
	Time since second vaccination (weeks)	First term	-0.089	0.029	0.002	0.12	0.56
		Second term	-0.015	0.017	0.35	>0.99	>0.99

Notes: B-Y: Benjamini-Yekutieli; H-B: Holm-Bonferroni; mRNA: messenger ribonucleic acid; SE: standard error.

Supplementary Table 6. Logistic regression model output for time trends in individual Long Covid symptoms and vaccination exposure variables

Outcome	Variable	Estimate	SE	P-value	Odds ratio (95% CI)
Difficulty concentrating	Time trajectory (per week)	0.010	0.005	0.051	1.010 (1.000 to 1.020)
	First vaccination (change in level)	-0.049	0.061	0.42	0.953 (0.846 to 1.073)
	Second vaccination (change in level)	-0.047	0.050	0.35	0.954 (0.866 to 1.052)
	Time since first vaccination (per week)	-0.002	0.008	0.81	0.998 (0.983 to 1.014)
	Time since second vaccination (per week)	-0.008	0.008	0.29	0.992 (0.977 to 1.007)
Fatigue	Time trajectory (per week)	0.001	0.004	0.77	1.001 (0.994 to 1.009)
	First vaccination (change in level)	-0.072	0.046	0.12	0.930 (0.849 to 1.019)
	Second vaccination (change in level)	-0.102	0.040	0.01	0.903 (0.835 to 0.976)
	Time since first vaccination (per week)	0.006	0.007	0.39	1.006 (0.993 to 1.019)
	Time since second vaccination (per week)	-0.011	0.006	0.08	0.989 (0.977 to 1.001)
Headache	Time trajectory (per week)	-0.004	0.005	0.46	0.996 (0.987 to 1.006)
	First vaccination (change in level)	-0.089	0.062	0.15	0.914 (0.810 to 1.033)
	Second vaccination (change in level)	-0.094	0.053	0.08	0.910 (0.819 to 1.010)
	Time since first vaccination (per week)	0.010	0.008	0.21	1.011 (0.994 to 1.027)
	Time since second vaccination (per week)	-0.020	0.008	0.02	0.980 (0.964 to 0.996)
Loss of smell	Time trajectory (per week)	0.002	0.005	0.73	1.002 (0.993 to 1.011)
	First vaccination (change in level)	-0.134	0.055	0.02	0.875 (0.785 to 0.975)
	Second vaccination (change in level)	-0.091	0.049	0.06	0.913 (0.829 to 1.005)
	Time since first vaccination (per week)	0.009	0.008	0.22	1.009 (0.994 to 1.025)
	Time since second vaccination (per week)	-0.016	0.008	0.04	0.984 (0.969 to 0.999)
Loss of taste	Time trajectory (per week)	-0.005	0.005	0.32	0.995 (0.985 to 1.005)
	First vaccination (change in level)	-0.097	0.063	0.13	0.908 (0.802 to 1.027)
	Second vaccination (change in level)	-0.094	0.052	0.07	0.910 (0.821 to 1.008)
	Time since first vaccination (per week)	0.018	0.008	0.03	1.019 (1.002 to 1.036)
	Time since second vaccination (per week)	-0.019	0.008	0.02	0.981 (0.965 to 0.997)
Memory loss or confusion	Time trajectory (per week)	0.014	0.006	0.01	1.014 (1.003 to 1.025)
	First vaccination (change in level)	-0.026	0.070	0.71	0.974 (0.850 to 1.117)
	Second vaccination (change in level)	0.029	0.055	0.60	1.029 (0.924 to 1.147)
	Time since first vaccination (per week)	-0.007	0.009	0.41	0.993 (0.976 to 1.010)
	Time since second vaccination (per week)	-0.009	0.009	0.31	0.991 (0.975 to 1.008)
Muscle ache	Time trajectory (per week)	0.006	0.005	0.23	1.006 (0.996 to 1.016)
	First vaccination (change in level)	-0.009	0.062	0.88	0.991 (0.878 to 1.118)
	Second vaccination (change in level)	-0.054	0.049	0.28	0.948 (0.860 to 1.044)
	Time since first vaccination (per week)	-0.001	0.008	0.88	0.999 (0.983 to 1.015)
	Time since second vaccination (per week)	-0.009	0.008	0.27	0.991 (0.976 to 1.007)

Shortness of breath	Time trajectory (per week)	-0.001	0.005	0.90	0.999 (0.991 to 1.008)
	First vaccination (change in level)	-0.055	0.054	0.31	0.946 (0.851 to 1.052)
	Second vaccination (change in level)	0.012	0.048	0.81	1.012 (0.921 to 1.111)
	Time since first vaccination (per week)	-0.005	0.008	0.50	0.995 (0.979 to 1.010)
	Time since second vaccination (per week)	-0.002	0.008	0.76	0.998 (0.983 to 1.013)
Trouble sleeping	Time trajectory (per week)	0.000	0.005	0.95	1.000 (0.990 to 1.010)
	First vaccination (change in level)	-0.092	0.063	0.15	0.912 (0.806 to 1.033)
	Second vaccination (change in level)	-0.094	0.054	0.08	0.910 (0.818 to 1.012)
	Time since first vaccination (per week)	0.004	0.009	0.67	1.004 (0.987 to 1.021)
	Time since second vaccination (per week)	-0.003	0.008	0.69	0.997 (0.980 to 1.013)
Worry or anxiety	Time trajectory (per week)	0.008	0.005	0.15	1.008 (0.997 to 1.018)
	First vaccination (change in level)	-0.090	0.067	0.18	0.914 (0.802 to 1.042)
	Second vaccination (change in level)	-0.034	0.057	0.55	0.967 (0.865 to 1.080)
	Time since first vaccination (per week)	0.000	0.009	0.98	1.000 (0.983 to 1.018)
	Time since second vaccination (per week)	-0.010	0.009	0.27	0.990 (0.973 to 1.008)
At least 3 of 21 symptoms	Time trajectory (per week)	0.005	0.004	0.21	1.005 (0.997 to 1.014)
	First vaccination (change in level)	-0.067	0.052	0.20	0.935 (0.844 to 1.037)
	Second vaccination (change in level)	-0.072	0.045	0.11	0.931 (0.852 to 1.016)
	Time since first vaccination (per week)	-0.001	0.007	0.94	0.999 (0.985 to 1.014)
	Time since second vaccination (per week)	-0.008	0.007	0.25	0.992 (0.978 to 1.006)
At least 5 of 21 symptoms	Time trajectory (per week)	0.005	0.005	0.36	1.005 (0.995 to 1.015)
	First vaccination (change in level)	-0.039	0.063	0.54	0.962 (0.850 to 1.089)
	Second vaccination (change in level)	-0.018	0.052	0.73	0.982 (0.886 to 1.088)
	Time since first vaccination (per week)	-0.005	0.009	0.56	0.995 (0.979 to 1.012)
	Time since second vaccination (per week)	-0.004	0.008	0.62	0.996 (0.979 to 1.012)

Notes: CI: confidence interval; SE: standard error. Estimates and standard errors are on the logit scale. Odds ratios for ‘time since first/second vaccination’ represent modification of the time trajectory. Estimates and odds ratios are adjusted for age, sex, white or non-white ethnicity, region/country, area deprivation quintile group, health status, whether a patient-facing health or social care worker, whether hospitalised with acute COVID-19, and calendar time of infection.

Supplementary Figure 1. Time trajectories estimated from linear and restricted cubic spline fits from 12 to 60 weeks post-infection in the absence of vaccination

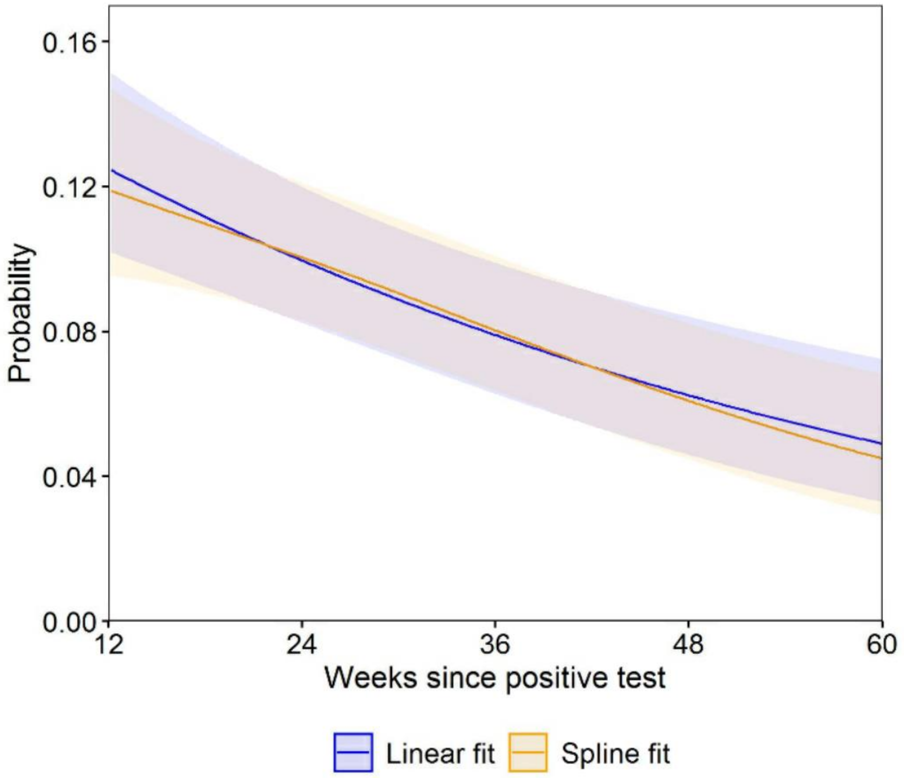

Notes: 60 weeks is the observed 95th percentile of time from infection to first vaccination in our sample. Probabilities are shown for a participant of approximately mean age (50 years) and in the modal group for other covariates (female, white, living in London, in an area in the least deprived quintile group, not a patient-facing health or social care worker, no pre-existing health conditions, not hospitalised at the acute phase of infection, and infected on 7 September 2020). While the estimated probabilities are specific to this profile, the proportional changes in probabilities over time since infection do not vary across characteristics and can therefore be generalised to other profiles. Shaded areas are 95% confidence intervals.

Supplementary Figure 2. Distribution of time between doses for the 84% of study participants who received two vaccinations before the end of the follow-up period

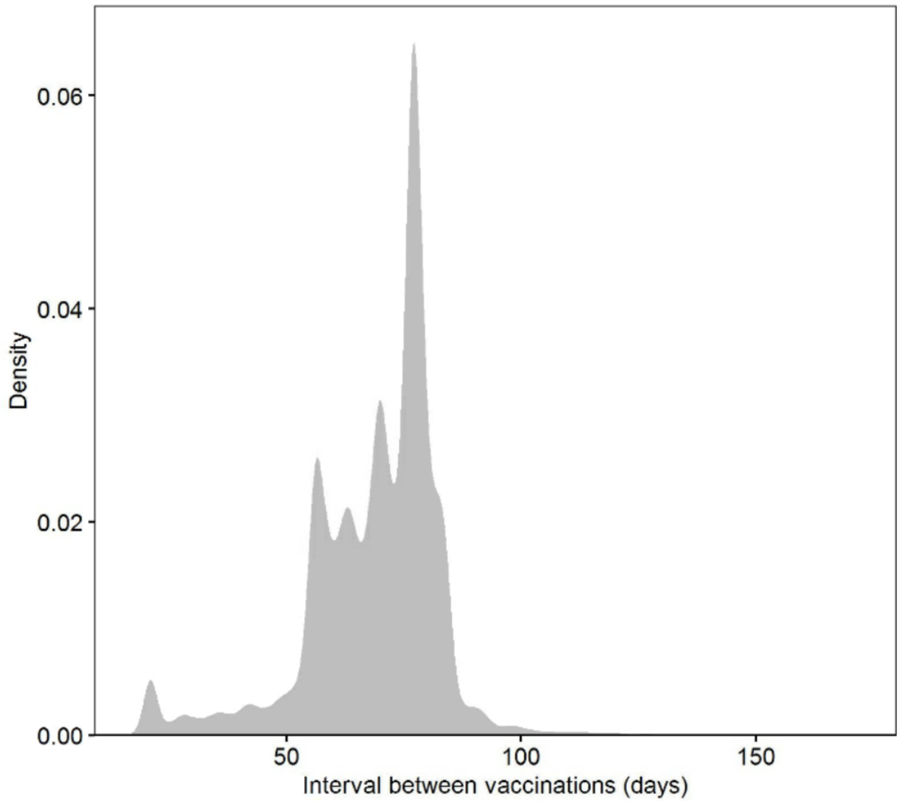

Supplementary Figure 3a. Modelled probabilities of Long Covid for an illustrative study participant, sensitivity analysis 1: participants with at least one observation before and after their first vaccination

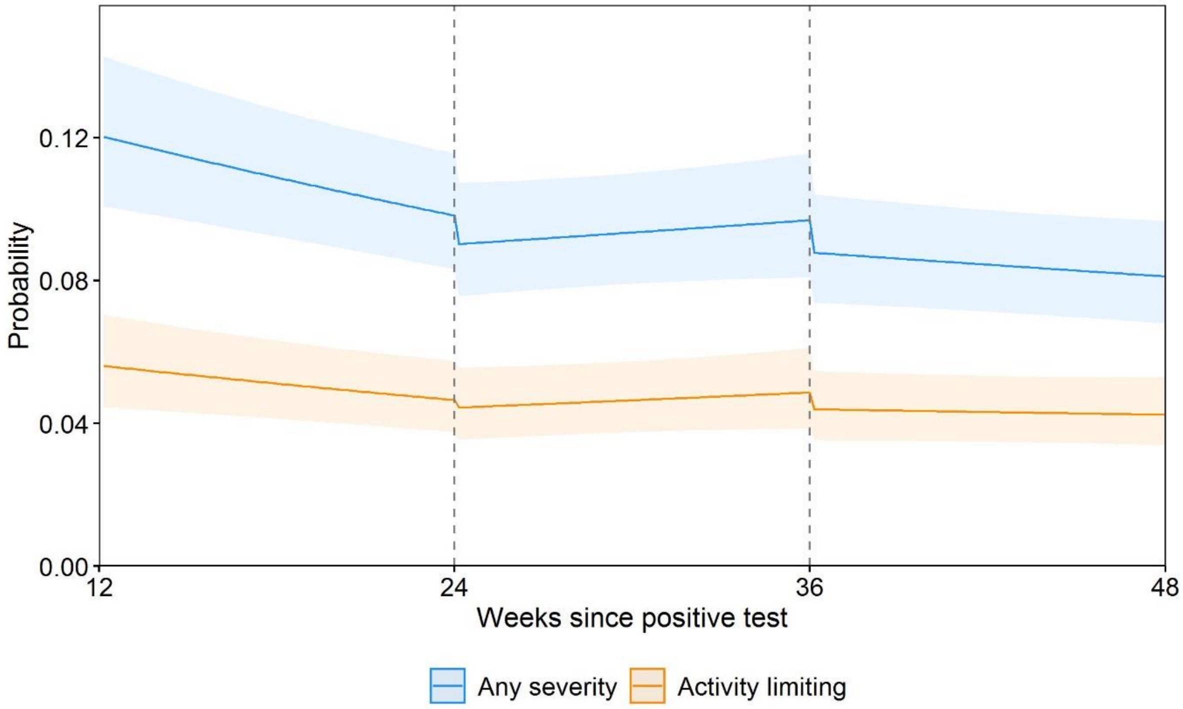

Notes: The sample comprised 12,971 participants with a mean age of 42 years and 10.8% with underlying health conditions (two of the main determinants of vaccination timing). Probabilities are shown for a participant aged 50 years and in the modal group for other covariates (female, white, living in London, in an area in the least deprived quintile group, not a patient-facing health or social care worker, no pre-existing health conditions, not hospitalised at the acute phase of infection, and infected on 7 September 2020). While the estimated probabilities are specific to this profile, the proportional changes in probabilities after vaccination do not vary across characteristics and can therefore be generalised to other profiles. Dashed lines indicate the timing of vaccination. Shaded areas are 95% confidence intervals.

Supplementary Figure 3b. Modelled probabilities of Long Covid for an illustrative study participant, sensitivity analysis 2: participants with at least one observation before and after their second vaccination

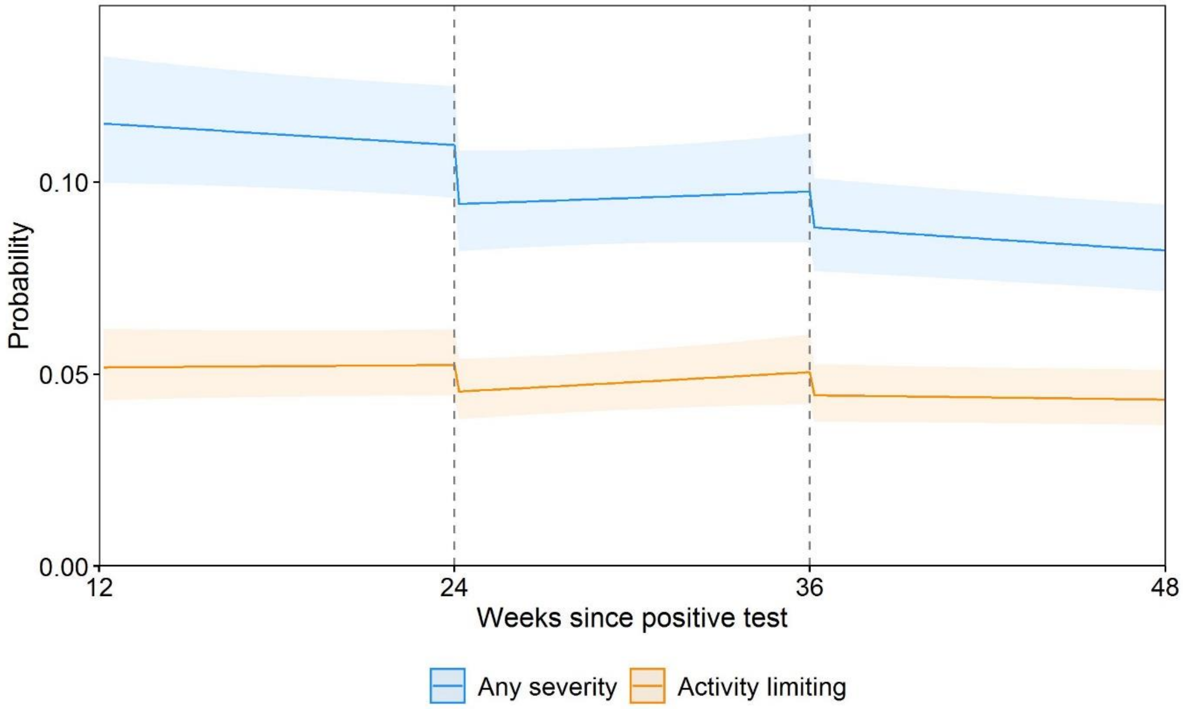

Notes: The sample comprises 20,335 participants with a mean age of 48 years and 14.0% with underlying health conditions (two of the main determinants of vaccination timing). Probabilities are shown for a participant aged 50 years and in the modal group for other covariates (female, white, living in London, in an area in the least deprived quintile group, not a patient-facing health or social care worker, no pre-existing health conditions, not hospitalised at the acute phase of infection, and infected on 7 September 2020). While the estimated probabilities are specific to this profile, the proportional changes in probabilities after vaccination do not vary across characteristics and can therefore be generalised to other profiles. Dashed lines indicate the timing of vaccination. Shaded areas are 95% confidence intervals.

Supplementary Figure 3c. Modelled probabilities of Long Covid for an illustrative study participant, sensitivity analysis 3: participants with at least three observations after their first vaccination

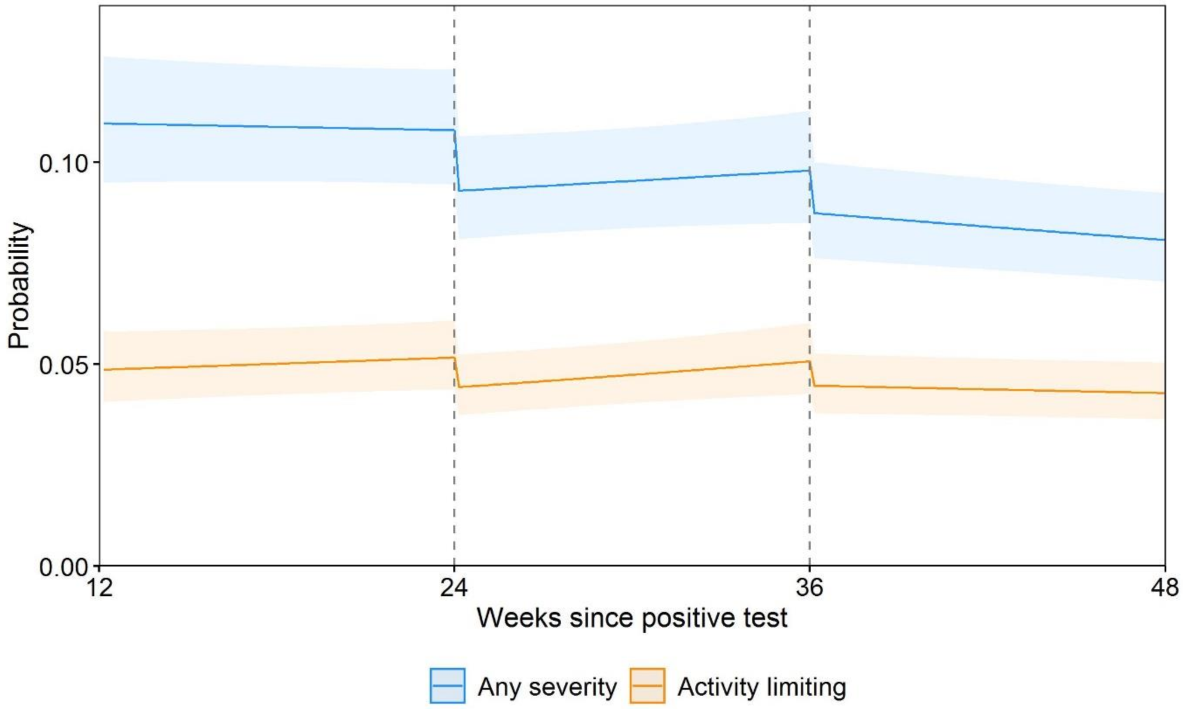

Notes: The sample comprises 20,635 participants with a mean age of 49 years and 15.1% with underlying health conditions (two of the main determinants of vaccination timing). Probabilities are shown for a participant aged 50 years and in the modal group for other covariates (female, white, living in London, in an area in the least deprived quintile group, not a patient-facing health or social care worker, no pre-existing health conditions, not hospitalised at the acute phase of infection, and infected on 7 September 2020). While the estimated probabilities are specific to this profile, the proportional changes in probabilities after vaccination do not vary across characteristics and can therefore be generalised to other profiles. Dashed lines indicate the timing of vaccination. Shaded areas are 95% confidence intervals.

Supplementary Figure 3d. Modelled probabilities of Long Covid for an illustrative study participant, sensitivity analysis 4: participants with at least three observations after their second vaccination

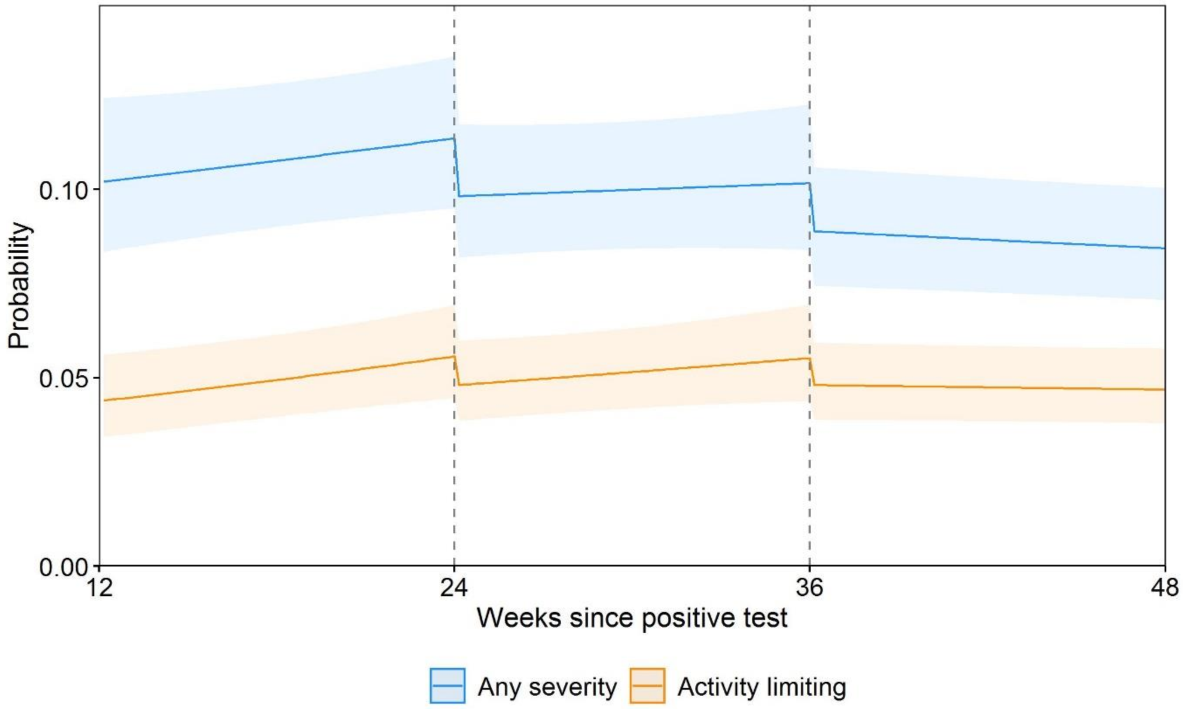

Notes: The sample comprises 12,288 participants with a mean age of 52 years and 18.0% with underlying health conditions (two of the main determinants of vaccination timing). Probabilities are shown for a participant aged 50 years and in the modal group for other covariates (female, white, living in London, in an area in the least deprived quintile group, not a patient-facing health or social care worker, no pre-existing health conditions, not hospitalised at the acute phase of infection, and infected on 7 September 2020). While the estimated probabilities are specific to this profile, the proportional changes in probabilities after vaccination do not vary across characteristics and can therefore be generalised to other profiles. Dashed lines indicate the timing of vaccination. Shaded areas are 95% confidence intervals.

Supplementary Figure 3e. Modelled probabilities of Long Covid for an illustrative study participant, sensitivity analysis 5: omitting follow-up visits within the first week after each vaccination

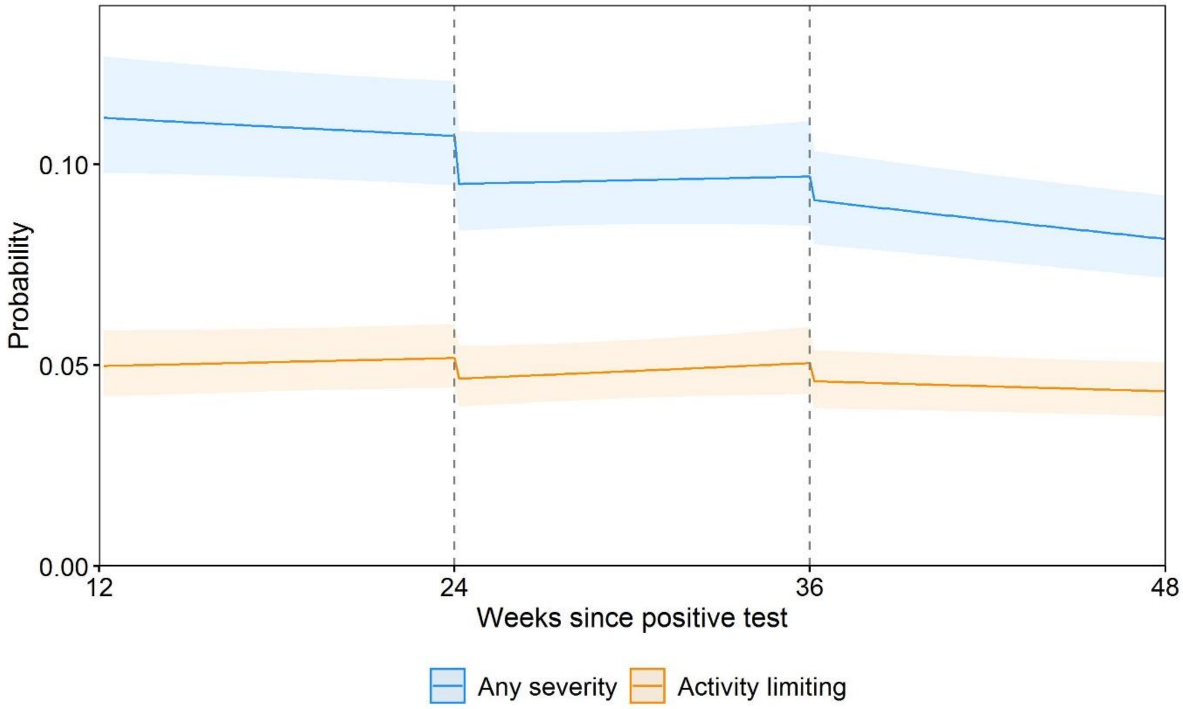

Notes: This analysis was based on 120,077 (89.6%) of the 133,965 follow-up visits used in the main analysis. Probabilities are shown for a participant aged 50 years and in the modal group for other covariates (female, white, living in London, in an area in the least deprived quintile group, not a patient-facing health or social care worker, no pre-existing health conditions, not hospitalised at the acute phase of infection, and infected on 7 September 2020). While the estimated probabilities are specific to this profile, the proportional changes in probabilities after vaccination do not vary across characteristics and can therefore be generalised to other profiles. Dashed lines indicate the timing of vaccination. Shaded areas are 95% confidence intervals.

Supplementary Figure 3f. Modelled probabilities of Long Covid for an illustrative study participant, sensitivity analysis 6: including participants who remained unvaccinated by their last follow-up visit during the study period

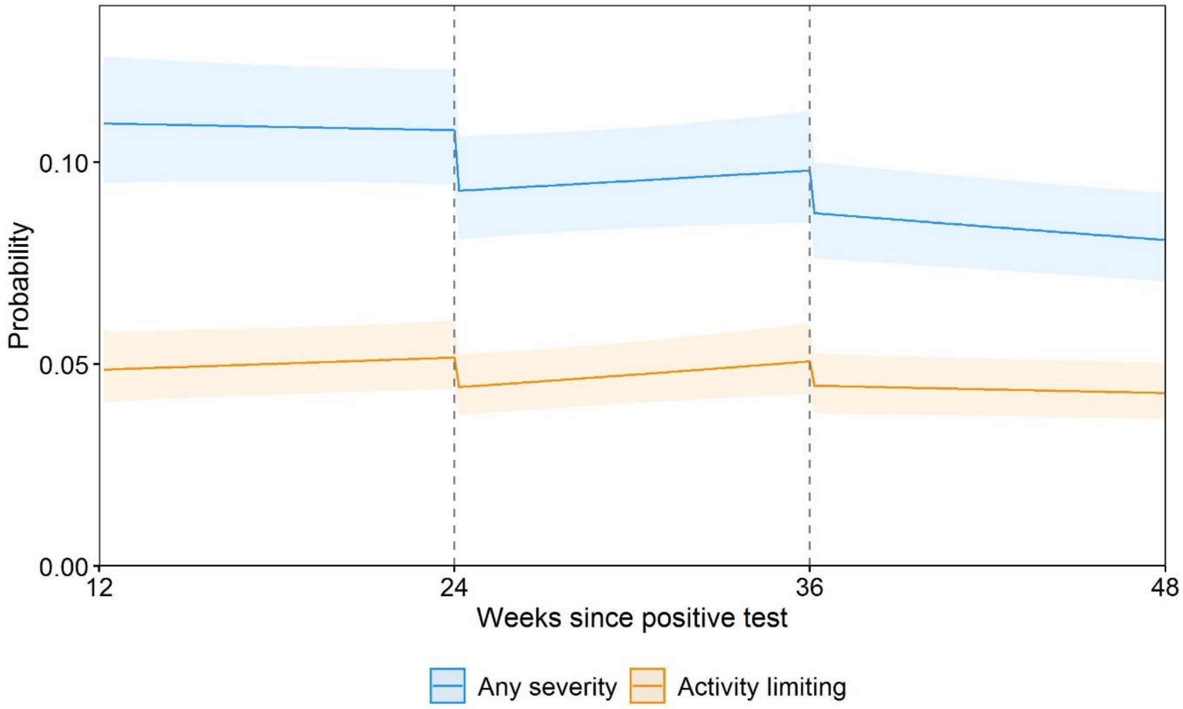

Notes: This analysis was based on 31,663 participants (compared with 28,356 in the main analysis). Probabilities are shown for a participant aged 50 years and in the modal group for other covariates (female, white, living in London, in an area in the least deprived quintile group, not a patient-facing health or social care worker, no pre-existing health conditions, not hospitalised at the acute phase of infection, and infected on 7 September 2020). While the estimated probabilities are specific to this profile, the proportional changes in probabilities after vaccination do not vary across characteristics and can therefore be generalised to other profiles. Dashed lines indicate the timing of vaccination. Shaded areas are 95% confidence intervals.

Supplementary Figure 3g. Modelled probabilities of Long Covid for an illustrative study participant, sensitivity analysis 7: excluding participants infected in the first wave of the pandemic

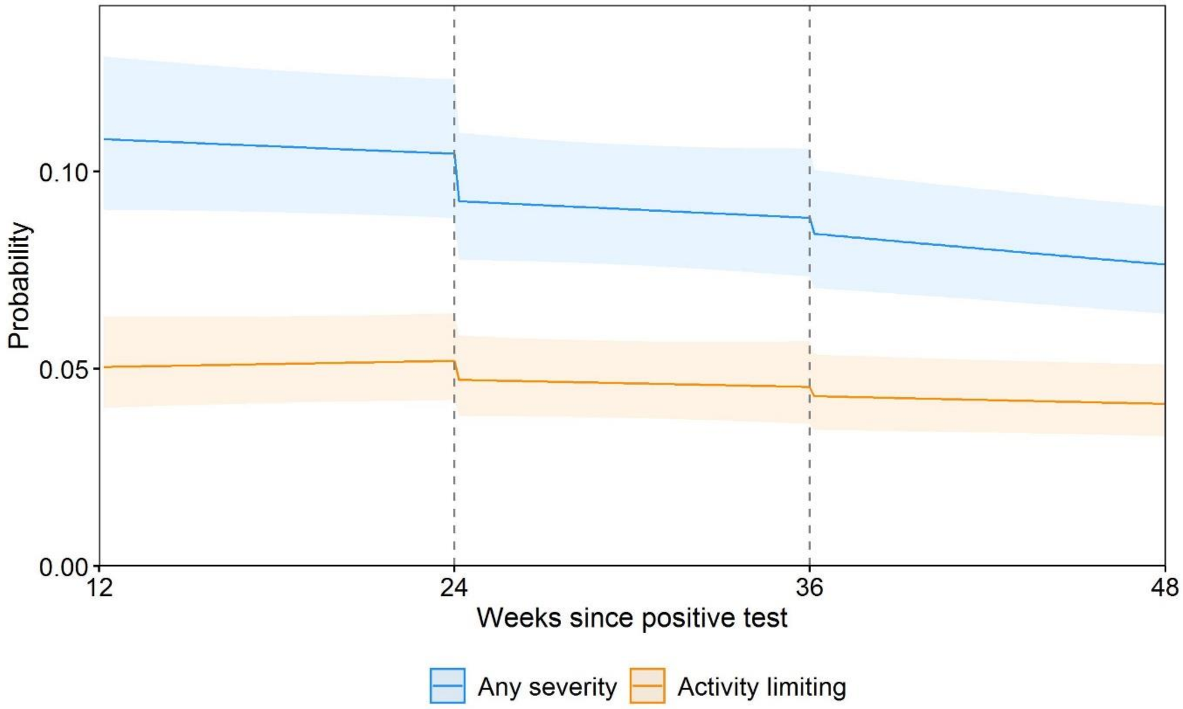

Notes: This analysis was based on 19,085 participants (compared with 28,356 in the main analysis) infected from 11 September 2020 onwards. Probabilities are shown for a participant aged 50 years and in the modal group for other covariates (female, white, living in London, in an area in the least deprived quintile group, not a patient-facing health or social care worker, no pre-existing health conditions, not hospitalised at the acute phase of infection, and infected on 7 September 2020). While the estimated probabilities are specific to this profile, the proportional changes in probabilities after vaccination do not vary across characteristics and can therefore be generalised to other profiles. Dashed lines indicate the timing of vaccination. Shaded areas are 95% confidence intervals.

Supplementary Figure 3h. Modelled probabilities of Long Covid for an illustrative study participant, sensitivity analysis 8: infection date for participants whose time of infection was determined by symptom onset >14 days before a positive swab reset to date of first positive swab

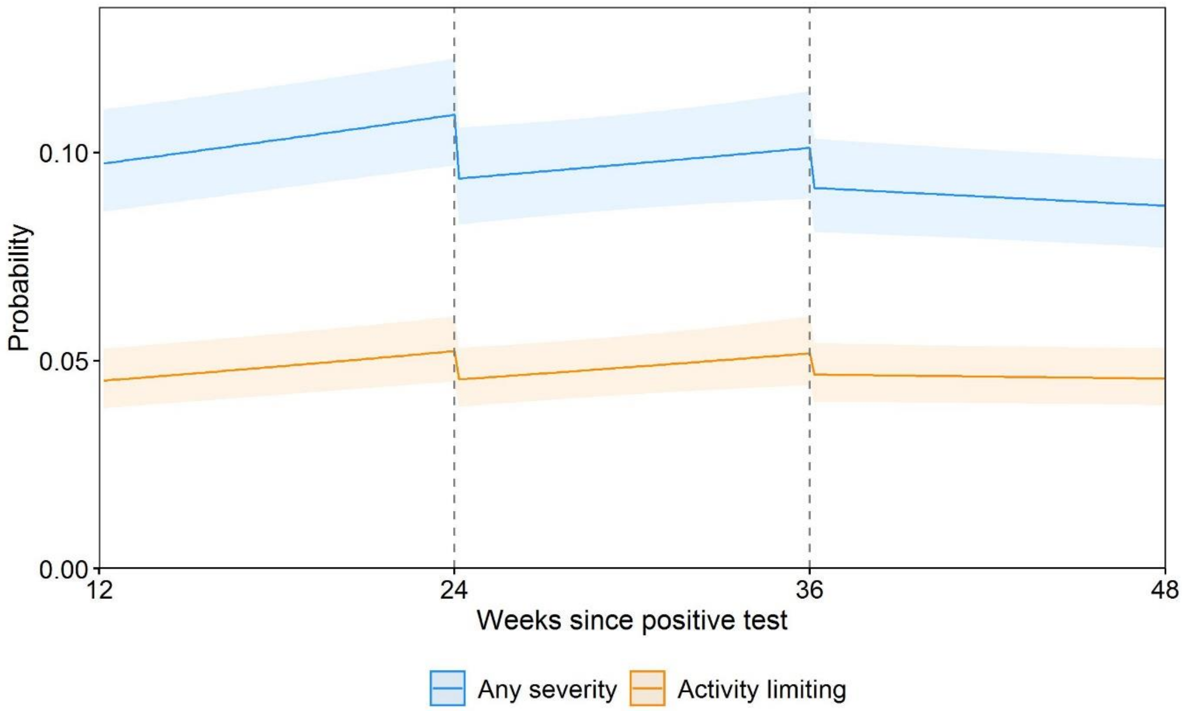

Notes: The infection date was moved forward by a median of 249 days among 698 participants (2.5%) whose time of infection was determined by symptom onset (rather than a confirmatory test) and was >14 days before a positive swab. Probabilities are shown for a participant aged 50 years and in the modal group for other covariates (female, white, living in London, in an area in the least deprived quintile group, not a patient-facing health or social care worker, no pre-existing health conditions, not hospitalised at the acute phase of infection, and infected on 7 September 2020). While the estimated probabilities are specific to this profile, the proportional changes in probabilities after vaccination do not vary across characteristics and can therefore be generalised to other profiles. Dashed lines indicate the timing of vaccination. Shaded areas are 95% confidence intervals.

Supplementary Figure 3i. Modelled probabilities of Long Covid for an illustrative study participant, sensitivity analysis 9: excluding participants whose infection date was determined by a positive blood test for SARS-CoV-2 antibodies that was obtained before or on the date of their first COVID-19 Infection Survey follow-up visit

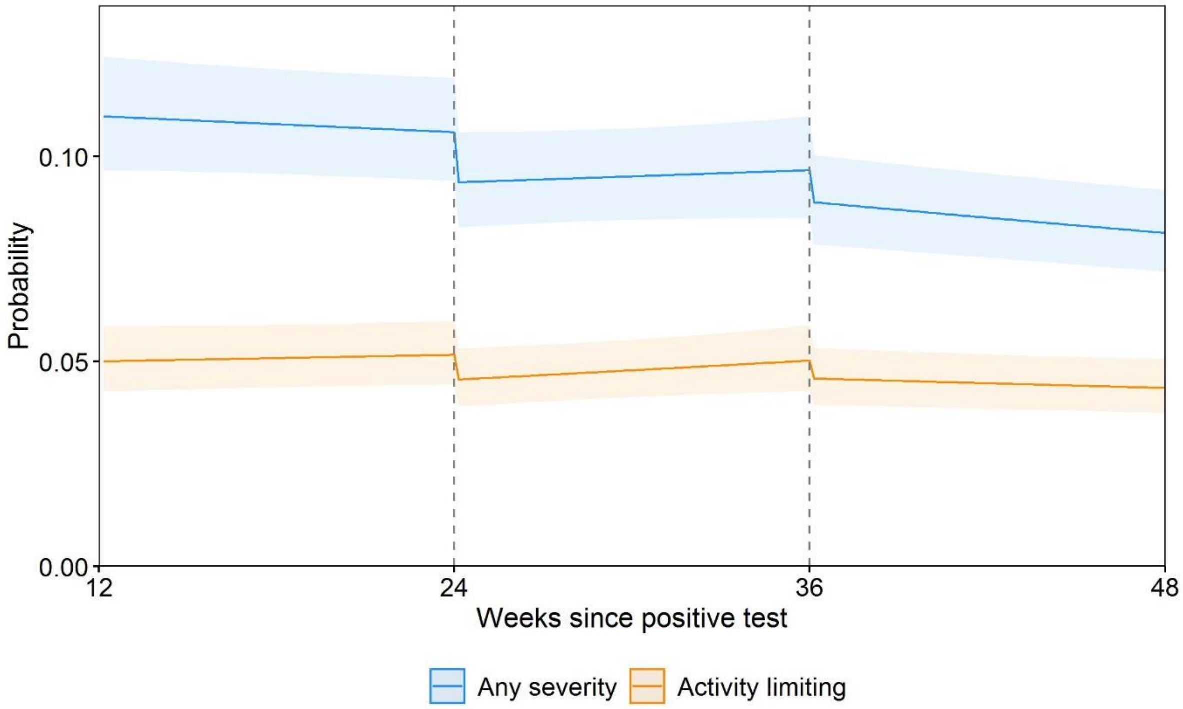

Notes: This analysis was based on 28,083 participants (compared with 28,356 in the main analysis). Probabilities are shown for a participant aged 50 years and in the modal group for other covariates (female, white, living in London, in an area in the least deprived quintile group, not a patient-facing health or social care worker, no pre-existing health conditions, not hospitalised at the acute phase of infection, and infected on 7 September 2020). While the estimated probabilities are specific to this profile, the proportional changes in probabilities after vaccination do not vary across characteristics and can therefore be generalised to other profiles. Dashed lines indicate the timing of vaccination. Shaded areas are 95% confidence intervals.

Supplementary Figure 4a. Modelled probabilities of Long Covid of any severity for illustrative study participants who received their first vaccination 6, 9, 12, and 15 months after infection

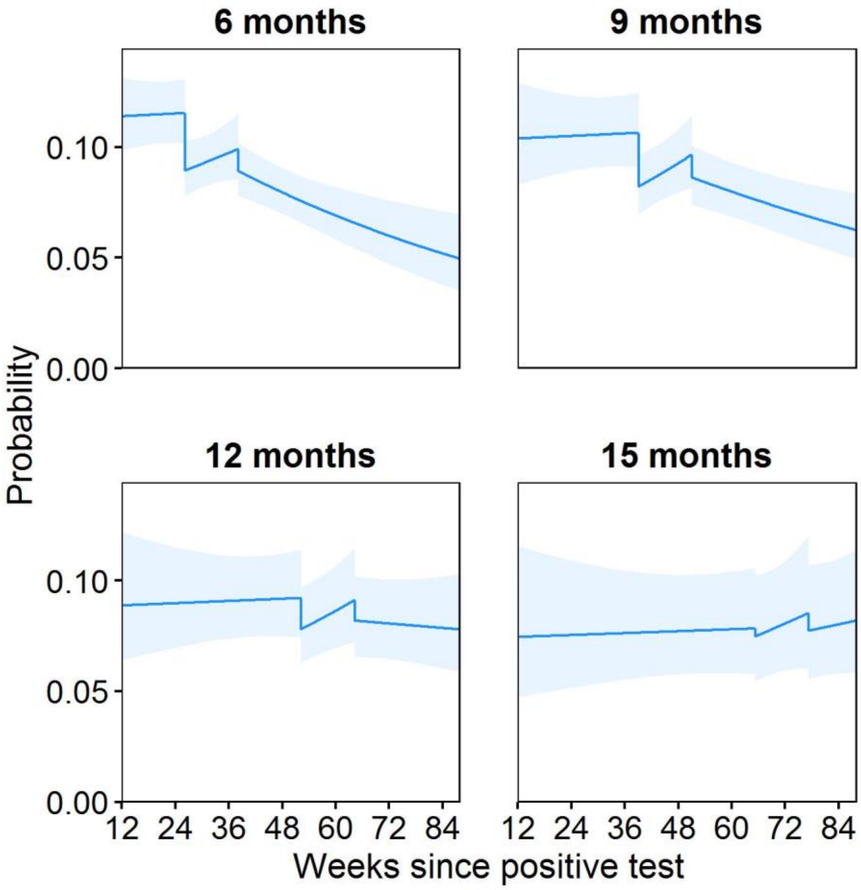

Notes: Probabilities are shown for a participant of approximately mean age (50 years) and in the modal group for other covariates (female, white, living in London, in an area in the least deprived quintile group, not a patient-facing health or social care worker, no pre-existing health conditions, not hospitalised at the acute phase of infection, and infected on 7 September 2020). While the estimated probabilities are specific to this profile, the proportional changes in probabilities after vaccination do not vary across characteristics and can therefore be generalised to other profiles. Dashed lines indicate the timing of vaccination. Shaded areas are 95% confidence intervals. Probabilities were obtained by interacting all four exposure variables (changes in level and slope after each dose) with duration from infection to first vaccination (modelled as a restricted cubic spline).

Supplementary Figure 4b. Modelled probabilities of activity-limiting Long Covid for illustrative study participants who received their first vaccination 6, 9, 12, and 15 months after infection

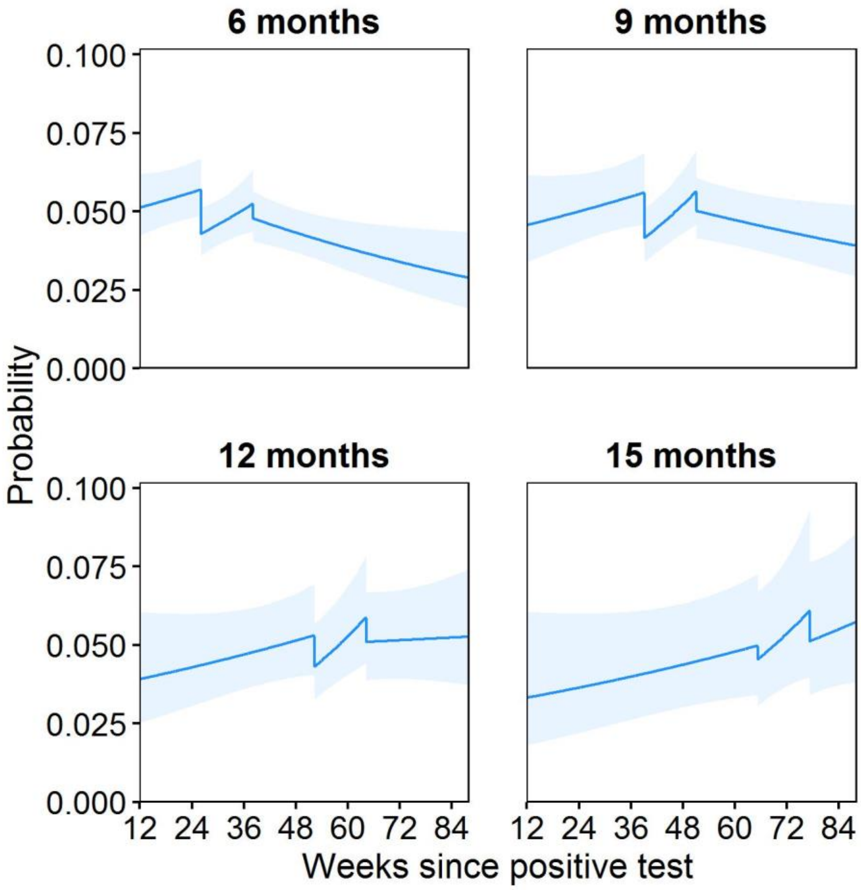

Notes: Probabilities are shown for a participant of approximately mean age (50 years) and in the modal group for other covariates (female, white, living in London, in an area in the least deprived quintile group, not a patient-facing health or social care worker, no pre-existing health conditions, not hospitalised at the acute phase of infection, and infected on 7 September 2020). While the estimated probabilities are specific to this profile, the proportional changes in probabilities after vaccination do not vary across characteristics and can therefore be generalised to other profiles. Dashed lines indicate the timing of vaccination. Shaded areas are 95% confidence intervals. Probabilities were obtained by interacting all four exposure variables (changes in level and slope after each dose) with duration from infection to first vaccination (modelled as a restricted cubic spline).

Supplementary Figure 5. Distribution of calendar time of first vaccination according to Long Covid status at the previous follow-up visit, stratified by age group

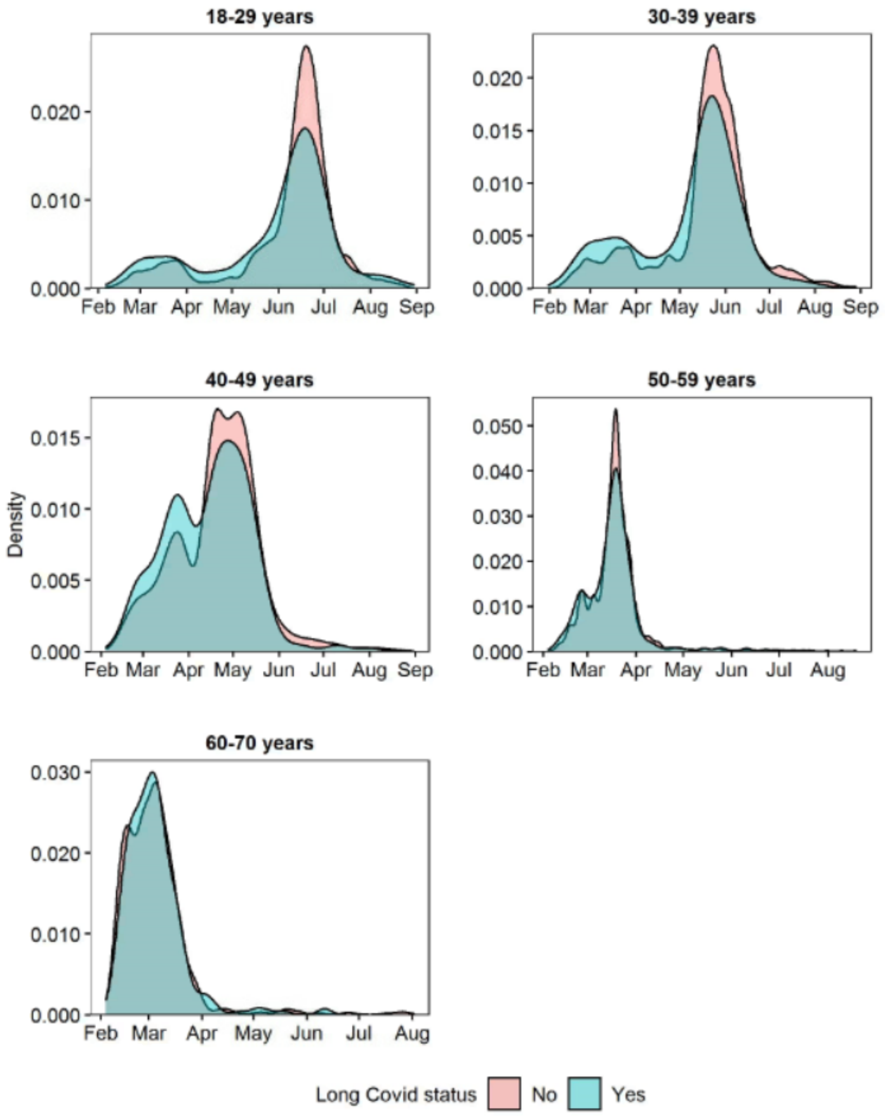

Notes: Densities estimated among 12,971 participants who received their first COVID-19 vaccination during the follow-up period.
